# Naphthoquinone Derivatives from *Angustimassarina populi* CF-097565 Display Anti-Tumour Activity in 3D Cultures of Breast Cancer Cells

**DOI:** 10.3390/molecules29020425

**Published:** 2024-01-15

**Authors:** Thomas A. Mackenzie, Fernando Reyes, Marta Martínez, Víctor González-Menéndez, Isabel Sánchez, Olga Genilloud, José R. Tormo, María C. Ramos

**Affiliations:** Fundación MEDINA, Av. Conocimiento 34, Health Sciences Technology Park, 18016 Granada, Spain; thomas.mackenzie@medinaandalucia.es (T.A.M.); fernando.reyes@medinaandalucia.es (F.R.); marta.martinez@medinaandalucia.es (M.M.); victor.gonzalez@medinaandalucia.es (V.G.-M.); isabel.sanchez@medinaandalucia.es (I.S.); olga.genilloud@medinaandalucia.es (O.G.)

**Keywords:** natural products, bioassay-guided fractionation, naphthoquinone, breast cancer, 3D culture

## Abstract

Cancer is one of the leading causes of death worldwide, with breast cancer being the second cause of cancer-related mortality among women. Natural Products (NPs) are one of the main sources for drug discovery. During a screening campaign focused on the identification of extracts from Fundación MEDINA’s library inhibiting the proliferation of cancer cell lines, a significant bioactivity was observed in extracts from cultures of the fungus *Angustimassarina populi* CF-097565. Bioassay-guided fractionation of this extract led to the identification and isolation of herbarin (**1**), 1-hydroxydehydroherbarin (**4**) plus other three naphthoquinone derivatives of which **3** and **5** are new natural products and **2** is herein described from a natural source for the first time. Four of these compounds (**1**, **3**, **4** and **5**) confirmed a specific cytotoxic effect against the human breast cancer cell line MCF-7. To evaluate the therapeutic potential of the compounds isolated, their efficacy was validated in 3D cultures, a cancer model of higher functionality. Additionally, an in-depth study was carried out to test the effect of the compounds in terms of cell mortality, sphere disaggregation, shrinkage, and morphology. The cell profile of the compounds was also compared to that of known cytotoxic compounds with the aim to distinguish the drug mode of action (MoA). The profiles of **1**, **3** and **4** showed more biosimilarity between them, different to **5**, and even more different to other known cytotoxic agents, suggesting an alternative MoA responsible for their cytotoxicity in 3D cultures.

## 1. Introduction

Cancer is one of the leading causes of death worldwide. Its existing burden in terms of incidence and mortality is expected to grow rapidly due to increased life expectancy and lifestyle problems that boost the risk of cancer. Worldwide, female breast cancer has now surpassed lung cancer as the most frequently diagnosed cancer. An estimated 2.2 million new breast cancer cases were diagnosed in women in 2020 establishing female breast cancer as the fifth leading cause of death [1,2]. A wide arsenal of therapeutic approaches for breast cancer includes surgery, radiotherapy, targeted endocrine/molecular therapy and chemotherapy [3]; however, it still produces half a million deaths each year. One of the reasons for this high mortality is that most of the patients with advanced breast cancer develop resistance to treatment [4]. Malignant cells that survive primary treatment continue to evolve, presenting a resistant population to established treatments; thereby, new treatments are currently needed to fight against this disease.

Since ancient times, Natural Products (NPs) have been used as anti-infectives, anti-inflammatories, antioxidants, analgesics and anti-tumourals and many compounds derived from NPs are in clinical use. The use of plants in traditional medicine is well known, and throughout recent history, metabolites of microbial origin have had an extraordinary impact on the welfare of humanity. Antibacterial and antifungal antibiotics, immunosuppressants, anti-tumoural and hypolipidemic agents derived from compounds of microbial origin have increasingly been used in clinical practice over the past 60 years. There is an outstanding diversity of chemical structures that nature, and especially microorganisms, are able to produce due to millenniums of evolution. Since only a small amount of the world’s biodiversity has been evaluated for potential biological activity, many more useful natural lead compounds await discovery, the challenge being how to access this natural chemical diversity. In the last decades, despite the difficulty of finding novel scaffolds, an increasing number of research groups have dedicated numerous efforts to explore alternative microbial sources, such as extreme environments like geographical poles, tremendously arid deserts, volcanoes, and deep ocean trenches, which have become an extraordinarily rich source of new drugs.

In the past 30 years, the percentage of NP or NP-inspired New Chemical Entities (NCEs) has risen to approximately 50% of all drugs on the market, and beyond, up to 74%, in the anti-tumour field [5]. The validation and selection of primary screening assays are vital to guarantee a selection of extracts or molecules with relevant pharmacological action. In the 2000s, the traditional NP screening was transiently abandoned by pharmaceutical companies, mostly due to (i) a frequently redundant discovery of previously isolated compounds, (ii) the technical difficulties associated to the isolation of active constituents from the inherently complex matrixes of the extracts, (iii) the incompatibility of NP extracts with High Throughput Screening (HTS) campaigns due to a limited resolution of the classic activity-guided fractionation methods, and finally, (iv) the combination between structural complexity and a prominently low titer production of NPs, making it difficult to characterize a novel molecule of interest, and further implying a relatively low cost/profit balance. Nonetheless, recent advances in technology running on highly sensitive machinery have been successfully implemented in NP-based drug discovery workflows, facilitating the rapid identification of novel bioactive compounds and their structure elucidation, rewardingly returning a substantial pharmaceutical momentum to NPs over synthetic molecules [6]. Indeed, during the last two decades, a handful of NPs, or NP-derivatives, have shown activity against breast cancer in vitro and in vivo [7,8]. As examples of the usefulness of NPs as anticancer drugs used in treatments for chemotherapy and targeted therapy against breast cancer we can find: vinorelbine (vinca alkaloid) [9], paclitaxel (taxane) [10] and/or doxorubicin (anthracyclin) [11]. Anyhow, more research is needed to bridge such encouraging pre-clinical data towards a better quality of life for the patients.

**Figure 1:**
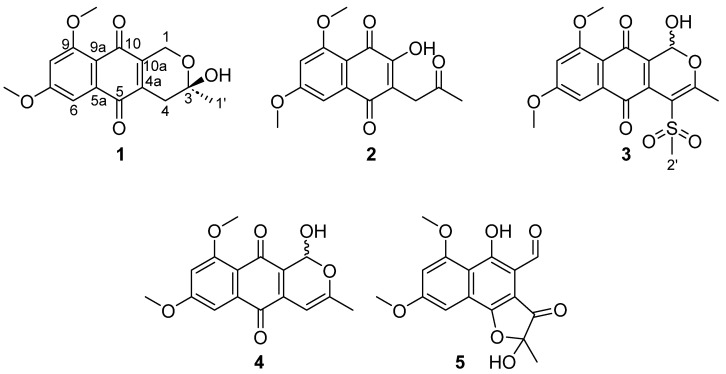
Structures of compounds **1**–**5**.

During a screening programme focused on the identification of active extracts from Fundación MEDINA’s Library of NPs of microbial origin inhibiting the proliferation of breast cancer cell lines, a significant bioactivity was observed in acetone extracts from cultures of the fungus *Angustimassarina populi* CF-097565. This genus, *Angustimassarina,* was introduced by Thambugala et al. in 2015 [12] to accommodate three species, being *A. populi* the type species. To date, thirteen species have been listed in the index fungorum (https://www.indexfungorum.org, accessed on 20 November 2023), all of them isolated as endophytes or epiphytes from different plant species [13,14,15,16]. Up to now, no specialized metabolites have been described from any of the members of this genus. This prompted us to study the bioactive extract of *A. populi* CF-097565 in depth. Its bioassay-guided fractionation led to the isolation of five naphthoquinone derivatives, (Figure 1), three of which are new. Four out of five (**1**, **3**, **4** and **5**) confirmed the growth inhibitory properties against the human breast cancer cell line MCF-7 without activity against other human cancer cell lines of different tissues like the pancreatic cell line MIA PaCa-2.

In order to give further therapeutic value to compounds of this family, their activity was tested in a cancer model of higher functionality such as the spheroid 3D cultures, in which an in-depth study of the effect of the set of compounds was carried out together with the comparison with other known cytotoxic agents.

## 2. Results

### 2.1. Structural Elucidation of New Natural Naphthoquinone Derivatives

Extracts from the fungus *Angustimassarina populi* CF-097565 were prioritized by its dose-dependent cytotoxic potency after a screening campaign with crude extracts from MEDINA’s Microbial NP Library with the breast cancer cell line MCF-7. A 3 L scaled-up fermentation broth of strain CF-097565 was extracted with acetone and subjected to chromatography on SP-207ss resin followed by preparative reversed-phase HPLC to obtain compounds **1**–**5** (Figure 1).

The molecular formula of C_16_H_16_O_6_ was assigned to compound **1** based on the presence of a protonated adduct in its (+)-ESI-TOF spectrum at *m*/*z* 305.1021 (calcd for C_16_H_17_O_6_^+^, 305.1020). ^1^H and HSQC NMR spectra (see SI), confirmed its identity as herbarin, a naphtoquinone antibiotic previously reported from several sources [17,18,19].

HRMS analysis of compound **2** established a molecular formula of C_15_H_14_O_6_, according to the presence in its spectrum of a protonated adduct at *m*/*z* 291.0892. Inspection of signals in its 1D and 2D NMR spectra established the presence in its structure of a naphtoquinone subunit, similar to that found in herbarin, but hydroxylated at C-10a together with the presence of additional signals of a chain containing a methylene, a ketone and a terminal methyl group attached at C-4a. All these data were in agreement with the structure of 3-hydroxy-5,7-dimethoxy-2-(2-oxopropyl)naphthalene-1,4-dione (2) depicted in Figure 1. This compound has previously been reported as a synthetic derivative of a pigment isolated from *Cylindrocarpon* sp. C.M.I. 127996 [20,21] and its isolation from a natural source is described herein for the first time. Full assignment of its NMR spectra is included in Table 1.

The molecular formula C_16_H_14_O_6_ determined for compound **4**, based on the presence of a [M + H]^+^ adduct at *m*/*z* 303.0867 in its (+)-ESI-TOF spectrum, had previously been reported for ascomycone A [22] and 1-hydroxydehydroherbarin [23]. Considering the close structural relationship between both compounds and herbarin isolated in our work, we recorded NMR spectra to confirm the structure of our compound. The two methoxy groups present in our molecule were placed in the aromatic ring as in 1-hydroxydehydroherbarin, based on their ^1^H NMR chemical shifts (*δ*_H_ 3.88 (9-OMe) and 3.92 (7-OMe), Table 1) compared to the chemical shifts in the ^1^H NMR spectrum of ascomycone A recorded in CDCl_3_ (*δ*_H_ 3.89 for the aromatic 7-OMe and 3.61 for the non-aromatic 1-OMe) [22]. Additional confirmation of the two methoxy groups at positions C-7 and C-9 of the aromatic ring were provided by intense HMBC correlations from the signals at (*δ*_H_ 3.88 and 3.92 to carbons at *δ*_C_ 161.4 (C-9) and 163.8 (C-7), respectively, confirming the structural difference with respect to ascomycone A. The structure of **4** as 1-hydroxydehydroherbarin (Figure 1). Its NMR spectra in DMSO recorded in this work are in good agreement with those previously published in acetone [18].

(+)-ESI-TOF analysis established a molecular formula of C_17_H_16_O_8_S for compound **3**, in agreement with the presence in its spectrum of a protonated adduct at *m*/*z* 381.0640. Their NMR spectra were quite similar to those of **4**, with the major differences being the absence of the signal at *δ*_H_ 5.93 in the spectrum of **4**, which was replaced by a methyl signal at *δ*_H_ 3.56 in **3**, together with the downfield shift of methyl C-1′ from *δ*_H_ 2.06 in **4** to 2.39 in **3**. Those changes, together with the differences in the molecular formula between both compounds, are in agreement with the replacement of H-4 in **4** by a methylsulphone in the structure of **3**. The downfield shifting of carbons C-3, C-4 and C-4a in **3** with respect to **4**, the chemical shift of C-2′, typical on methylsulphones [23] and an HMBC correlation between H_3_-2′ and C-4 confirmed this structural proposal. Additional support for the presence of a methylsulphone functionality in the molecule was obtained from the [M-CH_3_O_2_S+H]^+^ fragment at 302.0811 observed in the(+)-ESI-Tot spectrum of the molecule.

**Figure 2:**
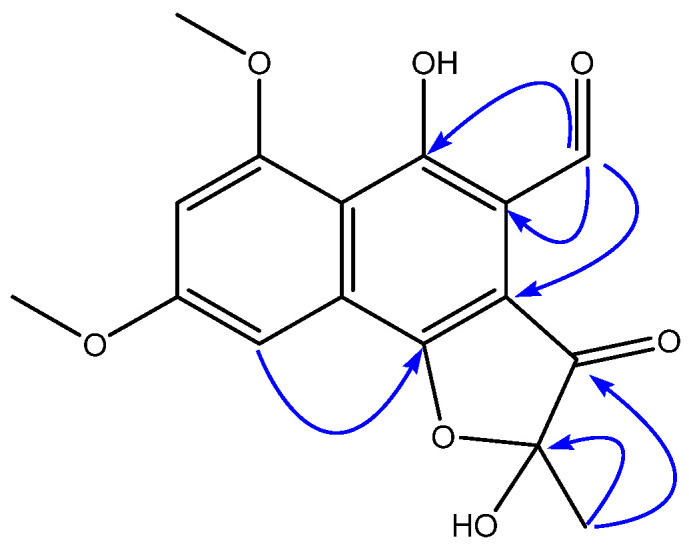
Key HMBC correlations for compound **5**.

A molecular formula of C_16_H_14_O_7_ was established for compound **5** based on (+)-ESI-TOF analysis and the presence of 16 signals in its ^13^C NMR spectrum. An inspection of its NMR spectra revealed the presence in its structure of a dimethoxylated aromatic ring, similar to that found in compounds **1**–**4**, but significant differences were found with the rest of the molecules. The two carbonyl signals present in the quinone moiety of **2**–**4** (Table 1) seemed to have been replaced by two oxygenated aromatic signals in the spectra of **5**, confirming a structural difference of this compound with the rest of the series. The presence of an aldehyde functionality seemed to be evident by the chemical shifts at this position (*δ*_H_ 10.45, *δ*_C_ 192.0). HMBC correlations from H-1 to C-4a, C-10, and C-10a (Figure 2) confirmed the placement of this aldehyde at C-10a. The ^1^H NMR signal for methyl H_3_-1′ was shielded in **5** with respect to **2**–**4**, and displayed HMBC correlations to carbons at *δ*_C_ 105.7 (C-3, hemiketal) and 198.9 (C-4, ketone). An additional HMBC correlation between H-6 at *δ*_H_ 7.10 and C-5 at *δ*_C_ 163.8 confirmed the presence of an oxygen at the latter and based on the molecular formula determined by HRMS, a ring closure via an ether bridge between C-3 and C-5 to form a 2-hydroxy-2-methylfuran-3(2*H*)-one moiety is proposed to complete the structure of **5** as depicted in Figure 1. Finally, although the absolute configuration at C-3 remains undetermined, the specific rotation measured for the compound indicates the existence of a pure enantiomer or an enantiomeric excess of one of the possible stereoisomers.

### 2.2. In Vitro Effectiveness of the Naphthoquinone Derivatives against 2D Breast Cancer Cells

**Figure 3:**
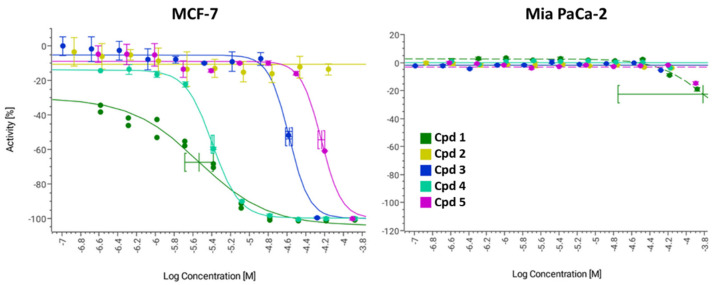
Dose–response curves of compounds (Cpd) **1**–**5** against MCF-7 and MIA PaCa-2 cell lines.

The set of five naphthoquinone derivatives **1**–**5**, identified as described above, were assayed for in vitro cytotoxicity against a human breast cancer cell line (MCF-7) and a human pancreatic cancer cell line (MIA PaCa-2), with the aim of checking the cancer-type specificity. The corresponding dose–response curves are shown in Figure 3 and ED_50_ (median Effective Dose) results are summarized in Table 2.

The most active compounds against breast cancer cells, with low micromolar ED_50_, were **1** and **4**; compound **3** resulted eight times less active than **1** and **4**, number **5** was the less active of the family (>50 µM), and compound **2** showed no activity. Moreover, none of the compounds showed activity against pancreatic cancer cells.

### 2.3. In Vitro Effects on 3D Breast Cancer Cells

In a broad sense, one of the bottlenecks causing the lack of efficiency in the discovery of novel cancer therapeutic agents comes from the extreme simplicity of the bidimensional (2D) tumour models classically used for lead validation and prioritization. These models present a shortage in physiological accuracy to recreate in vivo conditions, such as cell-cell interactions and oxygen gradients, nutrients, metabolites, and soluble signals. All of these factors contribute to the emergence of resistance mechanisms in a complex mixture of heterogeneous malignant cell populations against which chemotherapeutic agents have to present a sufficient level of potency and selectivity to make it through clinical trials [24]. Traditional 2D breast cancer models are not an exception for such a common trend due to their high simplicity, the poor cell interaction and specialization, and the lack of physiological environment [25,26]. In Vitro 3D tumour models (spheroids) are closer to real systems than 2D models. Spheroids allow you to better extrapolate the response to therapy in animal models and, to a certain extent, ensure its clinical feasibility with acceptable cost/time effectiveness and high ethical standards [27].

**Figure 4:**
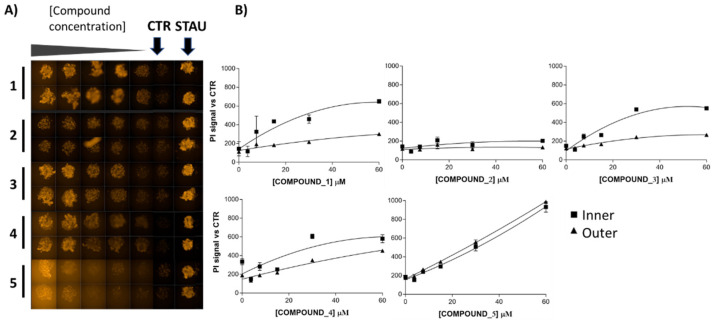

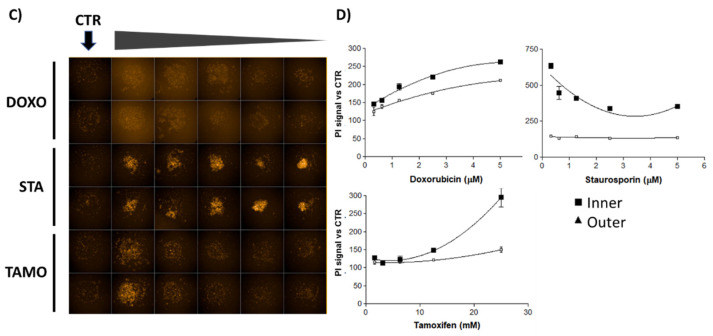
Dose–response curves of compounds against MCF-7 spheroids monitored by an imaging triple-staining method. (**A**) Confocal 3D images of cells spheroids stained by PI. Compounds were tested per duplicate in five points of 2-fold dose–response curves from 60 µM for 96 h of treatment. Staurosporine at 5 µM is used as positive control of cell death. (**B**) Quantification of the inner and outer sphere PI signals for each compound. (**C**) Confocal 3D images of treatments with cytotoxic compounds doxorubicin (DOXO), staurosporine (STA) and tamoxifen (TAMO) standards. Compounds were tested in five points of 2-fold dose–response curves from 5 µM, 5 µM and 25 µM respectively, for 96 h. (**D**) Quantification of the inner and outer sphere PI signals for each cytotoxic standard.

Consequently, we decided to test compounds **1**–**5** in MCF-7 spheroids in dose–response curves starting from 20 µg/mL where results were monitored by a triple-staining method (live cells-calcein AM, dead cells-propidium iodide (PI), nucleus-hoechst) [28] in a High-Content Imaging Operetta CLS System (PerkinElmer^®^, Waltham, MA, USA) to perform the corresponding image-based analyses. Evaluating the different fluorescence signals, the PI signal was the best dye to observe the effect of the compounds on the spheroids, as shown in Figure 4. Furthermore, image-based assays generated additional information about the effect of the compounds in detail as compounds **1**, **3**, **4** and **5** showed an increasing cell death effect, as it is shown with confocal 3D images projected in their 2D sections in Figure 4A, where further quantification of the inner- and outer-sphere PI signals are shown in Figure 4B.

Compounds **1**, **3**, **4** and **5** exhibited higher IP signals than controls, confirming 2D culture results, with the 3D culture approach showing additional information, for instance, a different death profile for compound **5** versus the others. To gain insights into the different death profiles induced by the compounds and to establish comparisons with known cytotoxic agents, MCF-7 spheroids were treated in parallel with doxorubicin, staurosporine and tamoxifen. Dose–response curves were obtained (images shown in Figure 4C), and inner and outer PI signals were quantified in Figure 4D. Staurosporine showed increased inner PI signal at all concentrations assayed; doxorubicin showed dose–response increase of inner and outer PI signals, and tamoxifen displayed a dose–response increase of inner PI signal.

**Figure 5:**
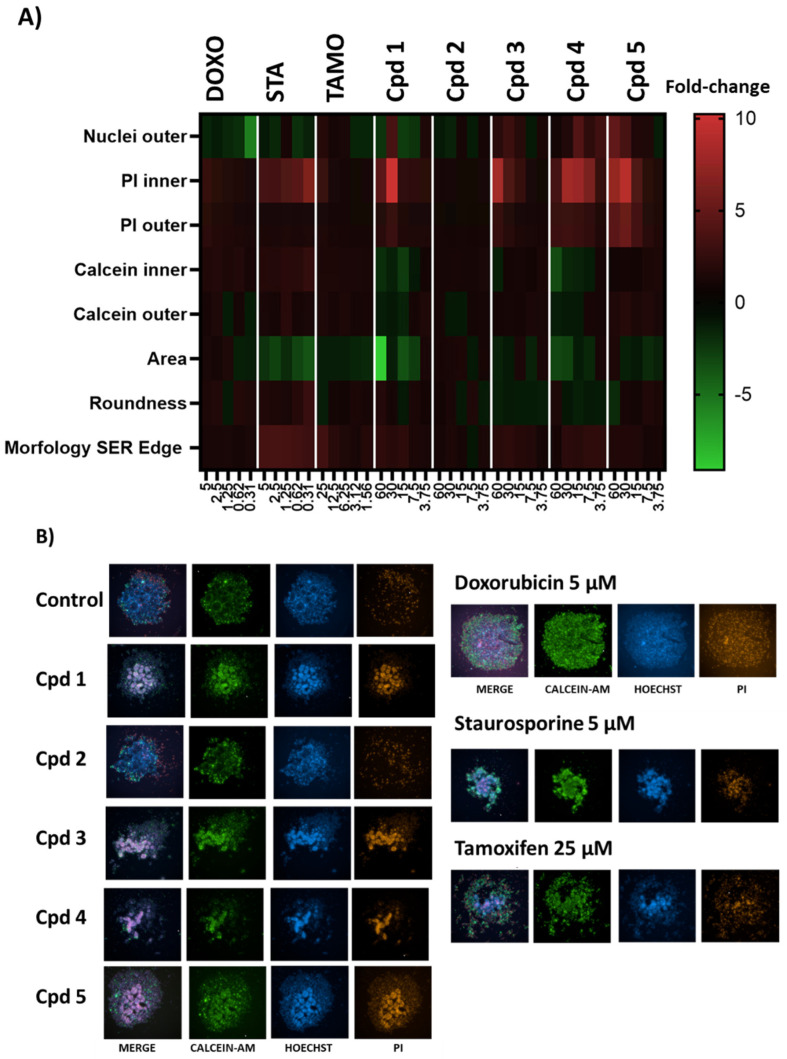
(**A**) Heatmap parameters comparison between known cytotoxic agents and purified compounds. Fold-changes of compounds, with regards to controls (DMSO 0.5%), for eight parameters (outer nuclei, inner PI, outer PI, inner calcein, outer calcein, area, roundness and morphology SER Edge) are shown for 96 h of treatment. Concentrations are shown in µM. (**B**) Images of spheroids treated with compounds at 30 µM and known compounds for 96 h of treatment. Triple-staining (live cells–calcein AM, dead cells–propidium iodide (PI), nucleus–hoechst) and merge is shown.

Taking advantage of the capabilities of the High-Content System, we considered that measurements of morphology, texture and fluorescence signals of treated spheroids might reveal other interesting parameters to compare profiles. Adapting this strategy to 3D cultures, the three dyes were used, and morphological and texture parameters were calculated. Finally, eight parameters were selected since they showed high consistency in the control replicates (DMSO 0.5%). Then, these parameters were compared for known cytotoxic agents and the naphthoquinone derivatives purified. A heatmap summarizing the results and the representative triple-staining images are shown in Figure 5.

In the case of the standards, the most remarkable changes for doxorubicin with regards to the controls (DMSO 0.5%) were the decrease in the number of outer nuclei (nuclei marked by hoechst in the outer part of spheroid, as indicative of spheroid disaggregation) and the increase of inner and outer PI signals (showing cell death). Staurosporine showed an increase of inner PI signal (cell death) and morphology SER Edge marker (that measures spots, edges and ridges and generates a relative quantification value, showing the complexity of the image morphology), with a decrease of the spheroid area (indicating shrinkage of the spheroid). Tamoxifen showed slight changes, decreasing the spheroid area, and dose–response increasing the morphology SER Edge.

**Figure 6:**
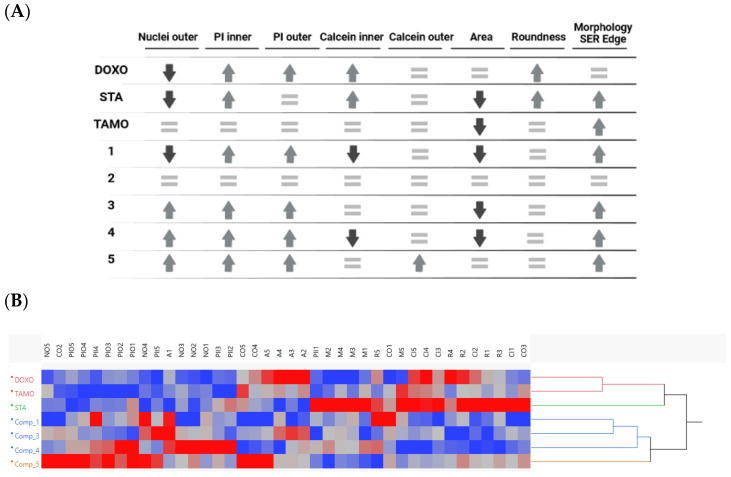
(**A**) Table showing the qualitative results of the cytotoxic agents and compounds **1**–**5**, compared to controls (DMSO 0.5%), in terms of the analysed parameters. Up-arrow, decrease >50% signal; down-arrow, increase >50% signal and =, no effect, for the maximal concentration assayed for each compound. Created with BioRender.com, accessed on 15 November 2023. (**B**) Hierarchical Cluster Analysis: dendrogram showing the biosimilarity between cytotoxic agents and active compounds. Blue, decreases; red, increases. NO, nuclei outer; PII, PI inner; PIO, PI outer; CI, calcein inner; CO, calcein outer; A, area; R, roundness; M, morphology SER Edge; numbered to indicate maximal to minimal concentration assayed for each compound (same as Figure 5A).

Compounds **1**, **3**, **4** and **5** showed an increase in the inner and outer PI signals and the morphology SER Edge, differing from each other for other parameters as shown in Figure 6A. On the contrary, compound **2** did not show changes in the measured parameters, confirming the results observed in 2D for no cell growth inhibition. Additionally, the biosimilarity between the three cytotoxic agents and our compounds is shown in the hierarchical cluster in Figure 6B.

On one hand, the absence of activity in compound **2** of the series suggests the requirement for the additional ring to the two already present in the naphthoquinone moiety for the compounds to exhibit the breast cancer cell growth inhibition biological activity. Moreover, in addition to showing specificity to breast cancer cells, compounds **1**, **3** and **4** displayed similar behaviour in MCF-7 spheroids, different to the one observed with compound **5**, whose structure shows significant differences with respect to the others. Specifically, the quinone moiety present in compounds **1**–**4** has been reduced in **5**, resulting in a hydroquinone moiety. Furthermore, a potential carbonyl group present at C-3 has undergone an attack by one the phenol groups of the hydroquinone located at C-5, leading to the formation of a 2-hydroxy-2-methylfuran-3(2*H*)-one moiety. Additionally, the presence of an aldehyde group at C-1 in **5** represents another distinctive structural feature in comparison to the rest of the compounds isolated. These differences in structure could explain that treatment with **5** increases the number of living cells (calcein positive), and of dead cells (PI) in the outer part of the spheroids, without decreasing their area, showing a disaggregation of the spheroid but without shrinkage, changes that are not observed for **1**, **2**, **3** or **4**.

On the other hand, DOXO and TAMO clustered differently with 60% of similarity among them, far from STA at 20%, and with almost no similarity (10%) among them and the purified compounds. Compounds **1** and **3** showed a similarity on their activity profiles of 50%, followed by **4** at 40% and **5** at 30% all of them in the same cluster. Resulting compounds **1**, **3** and **4** in a closer profile differentiated from compound **5**, and with all active purified compounds in a single cluster at a certain distance from the cytotoxic agents, which may indicate that the purified compounds have different mechanisms of action than the standards evaluated.

## 3. Discussion

The use of NPs as anti-tumour agents against breast cancer is fully proven [9,10,11] and advanced drug discovery of new therapeutic agents in this field involves the use of well-established cell lines, cancer stem cells, spheroids or organoids that mimic the patient’s tumour. Ideally, HTS assays should be quick, simple, and easy to implement, and capable of yielding robust results, preferably at a low cost. Great improvements have been implemented in vitro such as the use of 3D cultures and bioimaging in primary screenings, which add extra information compared to cytotoxicity screenings in classical 2D cell cultures. Culturing cells in 3D environments promotes the formation of multicellular tissues with proper cell–cell and cell–matrix interactions necessary for full functionality. This technology is starting to be successfully applied in cancer models [29,30]. Coupled to 3D cultures, high-content imaging systems have entailed huge advances in microscopy and image-based solutions [31]. Image acquisition using robotic fluorescent microscopy and automated image analyses have become essential tools in early drug discovery programs where high-content cellular imaging has increasingly met the challenges of high-throughput needs and starts to facilitate the integration of disease-relevant models and screens at early stages of drug discovery processes [32]. Hence, improvements in the discovery of NPs along with the emergence of these new technologies in cancer screening assays foresee the discovery of new and valuable drugs to reduce fatal breast cancer increase trend in forthcoming years.

Using these premises, we describe herein the identification, isolation, and structure elucidation of five compounds from the fungus *A. populi* CF-097565, four of them showing anti-tumour effects in 2D and 3D breast cancer models. These compounds were naphthoquinone derivatives, including the previously known herbarin, plus three new natural products, and a previously known compound described herein for the first time as a natural product. Naphthoquinones are a large group of aromatic compounds derived from naphthalene, spreading on a large scale in nature and are usually produced by bacteria, fungi, and plants. They exhibit a wide spectrum of biological activities, including anti-inflammatory, antifungal, anti-diabetic, antiviral, antiparasitic, antibacterial, anti-malarial, insecticidal, and cytotoxic effects [33,34].

In previous reports, some naphthoquinone derivatives were reported as cytotoxic against various cancer cell lines [35], and specifically in breast cancer [36], but to our knowledge, there are no reports describing the effect of naphthoquinone derivatives in cancer spheroid models. Furthermore, the use of high-content cellular imaging provided additional valuable information. In this sense, the measurements of morphology, texture and fluorescence signals of treated spheroids emerge as a very interesting tool to compare profiles looking for similarities, much the same as Cell Painting studies [37]. Cell Painting has been adopted for high throughput phenotypic screens with 2D cell cultures, but not yet with 3D cell cultures due to the complexity of the analysis. Adapting this strategy to the isolated compounds in spheroids, interesting results have been obtained.

Moreover the comparison of these compounds with known cytotoxic agents showed larger differences. Doxorubicin, a chemotherapeutic agent used to treat cancer, that acts by intercalating into DNA, and disrupting the topoisomerase-II-mediated DNA repair, generating free radicals that damage the cellular membranes, DNA and proteins, clustered in its activity profile far from the isolated naphthoquinones. In this sense, although doxorubicin is part of the anthraquinone family, which, like naphthoquinones, are structures based on 4-quinones, it seems that the small variances in the structures are reflected in a very different MoA. The same situation exists with staurosporine, an indolocarbazole used in preclinical research, that is an ATP-competitive kinase inhibitor with little selectivity. And, finally, also no concordances with the third cytotoxic standard compared, tamoxifen, a member of the triphenylethylene group of compounds, which acts as a selective estrogen receptor modulator (SERM) used to treat estrogen receptor-positive (ER+) breast cancer because it acts competing with 17β-estradiol at its receptor site (MCF-7 is an ER+ cell line).

According to Lee et al., 2019 [38], the MoA of several members of naphthoquinone-derivative family encompasses the induction of reduction–oxidation imbalance, alteration in mitochondrial transmembrane potential, Bcl-2 modulation, initiation of DNA damage, modulation of MAPK and STAT3 activity, alterations in the unfolded protein response and translocation of FOX-related transcription factors to the nucleus. This plethora of activities demonstrates the great versatility of the members of this family; thus, natural and synthetic, oligomeric substituted and heterocyclic naphthoquinone derivatives, can show promising biological activities against an extensive panel of diseases. Therefore, the identified new compounds of the fungus *Angustimassarina populi* may have selectivity on breast cancer cells with a different MoA whose identification will require further studies. Moreover, agents with similar structures to naphthoquinones have been approved for use by the FDA, such as mitomycin, a benzoquinone, drug approved for adult patients with low-grade upper tract urothelial cancer [39]. Altogether, data support that these isolated naphthoquinone derivatives are promising molecules with potential for the future developing of new therapeutic agents for breast cancer.

## 4. Materials and Methods

### 4.1. Cell Culture Lines and Reagents

Cell culture lines. Human breast cancer line, MCF-7 (7 (ATCC^®^ HTB-22^TM^, breast adenocarcinoma)) and pancreatic cancer cell line MIA PaCa-2 (ATCC^®^ CRL-1420^TM^, pancreas carcinoma) were purchased from ATCC (American Type Culture Collection, Manassas, VA, USA). MCF-7 was maintained in RPMI supplemented with 10% FBS, non-EAA and sodium pyruvate. MIA PaCa-2 was maintained in DMEM supplemented with 10% FBS, 2.5% FHS and 1% penicillin/streptomycin. Both cells were cultured in a humidified 37 °C incubator with 5% CO_2_.

Cell culture reagents. RPMI Media 1640 and Dulbecco’s Modified Eagle’s Medium (DMEM) were purchased from Biowest. Fetal bovine serum (FBS), non-EAA, sodium pyruvate, 2.5% FHS, penicillin–streptomycin and TrypLE Express were purchased from Gibco.

General Reagents. MMS (methyl methanesulfonate) was purchased from Sigma-Aldrich; PBS (phosphate buffered saline) from Lonza; DMSO from Merck; propidium iodide from Invitrogen Molecular Probes; Hoechst 33342 solution from Fischer Scientific; calcein-AM from BD Biosciences (Franklin Lakes, NJ, USA).

### 4.2. Strain Isolation and Identification

The fungal producer strain CF-097565 was isolated from living plant material of *Buxus sempervivens* collected in Pedralejos de las Truchas, Guadalajara (Spain) following a standard indirect technique for fungal endophyte isolation [40]. The axenic strain was preserved as frozen suspensions of septate mycelium in 10% glycerol at −80 °C. This strain is currently maintained in Fundación MEDINA’s Fungal Culture Collection (http://www.medinadiscovery.com, accessed 20 November 2023). DNA extraction, PCR amplification and DNA sequencing were performed as previously described [41]. Sequences of the complete ITS_1_-5.8S-ITS_2_ and initial LSU region or independent ITS and partial LSU rDNA were compared with those deposited at GenBank or the NITE Biological Resource Center (http://www.nbrc.nite.go.jp/, accessed 20 November 2023) by using the BLAST application [42,43]. Database matching with the ITS and partial LSU rDNA sequences (https://www.fungalbarcoding.org/, accessed 20 November 2023), yielded a complete sequence similarity (100%) to the holotype of *Angustimassarina populi* strain MFLUCC 13-0034 GenBank Accession Numbers KP899137 and KP888642), thus indicating that strain CF-097565 was genetically similar to *A. populi* and conspecific. High similar scores to other authentic fungal strain of this species, *A. populi* strain MFLUCC 17-1069 (GenBank Accession No. MF409170.1, 100% sequence similarity), indicated that CF-097565 could be classified as *Angustimassarina populi* [12].

### 4.3. Large-Scale Fermentation of Angustimassarina populi CF-097565

A. populi CF-097565 was cultured by inoculating ten mycelial agar plugs from yeast-malt agar culture into SMYA medium (Difco Laboratories^TM^ (Oxford, UK) neopeptone 10 g, maltose 40 g, Difco Laboratories^TM^ (Oxford, UK) yeast extract 10 g, agar 4 g and distilled water 1000 mL) in two flasks containing 50 mL of medium. The flasks were incubated on a rotary shaker at 220 rpm at 22 °C with 70% relative humidity. After growing the seed stage for 7 days, aliquots of 3 mL were used to inoculate 20 × 500 mL Erlenmeyer flasks containing the CYS80 production medium (Fisher Scientific^TM^, Waltham, MA, USA) S8600/70 sucrose 80 g, Sigma-Aldrich^TM^, St. Louis, MO, USA) C6304 corn meal yellow 50 g, Difco Laboratories^TM^ (Oxford, UK) yeast extract 1 g and 1000 mL of distilled water). The 20 seeded flasks were shaken at 220 rpm for 14 days at 22 °C and 70% relative humidity.

### 4.4. Chemical Instrumentation

Optical rotations were measured on a Jasco P-2000 polarimeter (JASCO Corporation, Tokyo, Japan). IR spectra were recorded with a JASCO FT/IR-4100 spectrometer equipped with a PIKE MIRacle single reflection ATR accessory. NMR spectra were recorded on a Bruker Avance III spectrometer (500 and 125 MHz for ^1^H and ^13^C NMR, respectively) equipped with a 1.7 mm TCI MicroCryoProbe (Bruker Biospin, Fällanden, Switzerland). Chemical shifts were reported in ppm using the signals of the residual solvents as internal reference (*δ*_H_ 2.50 and *δ*_C_ 39.51 for DMSO-*d*_6_). LC-UV-LRMS analysis were performed on an Agilent 1260 Infinity (Agilent Technologies, Waldbronn, Germany) single quadrupole LC-MS system as previously described [44]. ESI-TOF and MS/MS spectra were acquired using a Bruker maXis QTOF (Bruker Daltonik GmbH, Bremen, Germany) mass spectrometer coupled to an Agilent Rapid Resolution 1200 LC (Agilent Technologies). The mass spectrometer was operated in positive ESI mode. The instrumental parameters were 4 kV capillary voltage, drying gas flow of 11 L min^−1^ at 200 °C, and nebulizer pressure of 2.8 bar. TFA-Na cluster ions were used for mass calibration of the instrument prior to sample injection. Pre-run calibration was carried out by infusion with the same TFA-Na calibrant. Medium pressure liquid chromatography (MPLC) was performed on semiautomatic flash chromatograph (CombiFlash Rf400 Teledyne ISCO Inc., Lincoln, NE, USA) with a precast reversed-phase column. Semi-preparative HPLC separation was performed on Gilson GX-281 322H2 (Gilson Technologies, Middleton, WI, USA). Acetone used for extraction was analytical grade. Solvents employed for isolation were all HPLC grade.

### 4.5. Bioassay-Guided Isolation of Naphthoquinone Derivatives

A 3 L scaled-up fermentation broth of strain CF-097565 was extracted by adding acetone (3 L) and shaking at 220 rpm for 2 h in several 500 mL flasks. The extracted mycelium was separated by filtration in a Büchner at 7500 rpm, and the remaining acetone extract was concentrated under a N_2_ stream to a final volume of 2.5 L. This aqueous residue was loaded onto an SP-207ss resin column (70 g, 22 × 100 mm) and eluted with an acetone/H_2_O stepped gradient (10/90 for 6 min, 20/80 for 6 min, 40/60 for 6 min, 60/40 for 6 min, 80/20 for 6 min and 100/0 for 16 min, 20 mL/fraction) at 10 mL/min to give thirty 10 mL fractions.

Aliquots of each fraction were evaluated for MCF-7 cell growth inhibition and according to HPLC-MS-UV 210 nm analysis were pooled in two groups of active fractions F19 to F22 (807.7 mg) and F23 to F24 (264.9 mg). Pools were then subjected to preparative reversed-phase HPLC in sequential batches of 100–125 mg in a Zorbax SB-C8 column (21.2 × 250 mm, 7 μm; 20 mL/min; UV detection at 210 and 280 nm) applying a linear H_2_O:CH_3_CN gradient with 0.1% TFA (20–30%: 1–75 min, 30–100%: 75–76 min, 100%: 76–82 min), to afford 5 pure compounds at retention times of 31 min (**1**, 8.9 mg), 34 min (**2**, 2.0 mg), 39 min (**3**, 2.1 mg), 50–55 min (**4**, 78.1 mg) and 64–66 min (**5**, 6.3 mg). Purity of the compounds was determined over 89% by analytical reverse-phase HPLC-UV area at 210 nm.

Compound **1**: red amorphous solid; for ^1^H and HSQC NMR spectra, see supporting information; (+)-ESI-TOF MS *m*/*z* 305.1021 [M + H]^+^ (calcd for C_16_H_17_O_7_^+^, 305.1020), 287.0918 [M − H_2_O + H]^+^ (calcd for C_16_H_13_O_5_^+^, 287.0914).

Compound **2**: brown amorphous solid; for ^1^H and ^13^C NMR data see Table 1; (+)-ESI-TOF MS *m*/*z* 291.0892 [M + H]^+^ (calcd for C_15_H_15_O_6_^+^, 291.0863).

Compound **3**: brown amorphous solid; for ^1^H and ^13^C NMR data, see Table 1; (+)-ESI-TOF MS *m*/*z* 381.0640 [M + H]^+^ (calcd for C_17_H_17_O_8_S^+^, 381.0639), 363.0534 [M − H_2_O + H]^+^ (calcd for C_17_H_15_O_7_S^+^, 363.0533), 302.0811 [M − CH_3_O_2_S + H]^+^ (calcd for C_16_H_14_O_6_^+^, 302.0785).

Compound **4**: orange amorphous solid; for ^1^H and ^13^C NMR data, see Table 1; (+)-ESI-TOF MS *m*/*z* 303.0867 [M + H]^+^ (calcd for C_16_H_15_O_6_^+^, 305.0863), 285.0793 [M − H_2_O + H]^+^ (calcd for C_16_H_13_O_5_^+^, 285.0757).

Compound **5**: mustard-colored amorphous solid; ${\left[\alpha \right]}_{D}^{25}$ –10.1 (*c* 0.13, DMSO-*d_6_*); for ^1^H and ^13^C NMR data, see Table 1; (+)-ESI-TOF MS *m*/*z* 319.0820 [M + H]^+^ (calcd for C_16_H_15_O_7_^+^, 319.0812).

### 4.6. 2D Culture Cytotoxicity Assay: MTT Test

The tumour cell lines used were MCF-7 for screening and ED_50_ dose–response curves, and MIA PaCa-2 for ED_50_ dose–response curves. The extracts from MEDINA’s NP Library were assayed at a final dilution of 1/20 × whole-broth equivalents (WBE) for screening and maximum concentration of dose–response curves. The cytotoxicity of compounds **1**–**5** was tested in a MTT test starting at a concentration of 60 µM in 10-point 2-fold dose–response curves, in triplicate [45]. MTT test for primary screening was developed in 96-well plates and, for validation and ED_50_ dose–response curves, the procedure was improved through the use of 384-well plates and the Echo Acoustic Liquid Handling System (Echo^®^ 550, formerly owned by Labcyte^®^, San Jose, CA, USA) and now by Beckman-Coulter^®^, Brea, CA, USA)) that uses acoustic energy to dispense nanolitre-volumes of samples [46].

Briefly, cells were seeded at 10,000 cells/well in 96-well plates, and 4000 or 8000 cells/well in 384-well plates for MIA PaCa-2 and MCF-7 cells, respectively. After 24 h, cells were treated with extracts/compounds/controls and incubated at 37 °C, 95% humidity and 5% CO_2_ for 72 h. MMS 2 mM was used as positive control of cell death and DMSO 0.5% as negative control. For 384-well plates, the Echo^®^ was used for compound addition, whereas the Freedom EVOware (TECAN) was used for 96-well plates.

After the incubation period, the plates containing treated cells were washed with 100 µL/25 µL of PBS 1×. Then, MTT reagent (3-(4,5-Dimethyl-2-thiazolyl)-2,5-diphenyl-2H-tetrazolium bromide) (ACROS Organics^®^, Geel, Belgium) was added at 0.5 mg/mL and plates were incubated for 3 h. The corresponding supernatant was then removed and 100 µL/20 µL of DMSO 100% were added to each well in order to dissolve the resulting formazan precipitates. After shaking, absorbances at 570 nm were measured with an Envision^TM^ plate reader (PerkinElmer, Waltham, MA, USA). The resulting data were fitted to a standard ED_50_ model by Genedata Screener^®^ 18.0.4 Standard software.

### 4.7. Spheroid Formation and High Content Imaging

MCF-7 spheroid generation was carried out using 10 µL of a heated 1.5% *w*/*v* agarose solution dispended by liquid dispensers (Multidrop Combi^TM^, Thermo Scientific^®^ Waltham, MA, USA) into sterile 384-well clear bottom imaging plates (Greiner^®^ Kremsmünster, Austria, 384 well plate) and after gelation of agarose, 5000 cells were seeded in 30 µL of cell medium using a Multidrop [47]. Plates were incubated under standard cell culture conditions for 4 days to allow formation of reproducible spheroids. Then, compounds in nanolitre-volumes were added with the Echo^®^ for an additional 3 days.

Then, spheroids are stained with three-dyes staining method for 1 h with 1.3 µM Calcein AM (BD^®^ Biosciences, Franklin Lakes, NJ, USA), as stain for living cells and 10 µg/mL propidium iodide (Invitrogen), as stain for dead cells and 30 µM Hoechst 33342 (Life Technologies^®^, Carlsbad, CA, USA) as counter stain for all nuclei. Imaging of spheroids was carried out using Operetta^®^ CLS High-Content Analysis System (PerkinElmer^®^, Waltham, MA, USA) with 5× objective and Z-stack using 27 planes with steps of 20 µm. Then, maximum projection images of Z-stack were analysed using Harmony^TM^ 5.2 software (PerkinElmer^®^, Waltham, MA, USA). The resulting data were analysed using GraphPad^®^ Prism^TM^ 10, from Dotmatics, Boston, MA, USA) to generate dose–response curves and heatmap and JMP^TM^ 12 from JMP^®^, Cary, NC, USA (with Ward’s clustering method and linear distances depicted) to generate the similarities dendrogram among activity profiling.

## 5. Conclusions

A screening program focused on the identification of extracts from Fundación MEDINA’s Microbial NP Library inhibiting the proliferation of cancer cell lines, showed a significant bioactivity in extracts from cultures of *Angustimassarina populi* CF-097565. Bioassay-guided fractionation of a 3 L broth extract of this fungus led to the identification and isolation of five naphthoquinone derivatives, four of which (**1**, **3**, **4** and **5**) confirmed specific cytotoxic effect against human breast cancer cells in 2D and in 3D cultures.

Naphthoquinone compounds have been reported for a myriad of effects, demonstrating the great versatility of the members of this family and their promising biological activities against an extensive panel of diseases. To understand the potential of the compounds isolated, deeper bioimaging analyses on cell mortality, spheroid disaggregation, shrinkage, and morphology were performed and compared to known cytotoxic compounds of different MoA. Profiles obtained suggested alternative MoA to the ones of doxorubicin, staurosporine or tamoxifen, with **1**, **3** and **4** showing more biosimilarity among them than with **5** for their cytotoxicity in 3D cultures. Therefore, the identified compounds may have totally diverse mechanisms of action, whose identification will require further studies as potential molecules of interest for the future development of new therapeutic agents against breast cancer.

## Figures and Tables

**Table 1:** ^1^H NMR (500 MHz) and ^13^C NMR (125 MHz) data for **2**–**5** (in DMSO-*d*_6_).

	2		3		4		5	
Position	*δ*_H_, mult, *J*	*δ*_C_	*δ*_H_, mult, *J*	*δ*_C_	*δ*_H_, mult, *J*	*δ*_C_	*δ*_H_, mult, *J*	*δ*_C_
1	-	-	6.39, d, 6.0	88.3	6.32, s	87.8	10.45, s	192.0
1-OH	-		8.23, d, 6.4		7.50, brs		-	
3		204.4		169.8		159.8		105.7
4	3.51, s	37.7		115.5	5.93, s	92.3		198.9
4a		115.0		133.7		123.9		106.6
5		183.3		183.0		183.0		163.9
5a		135.9		136.4		134.8		129.1
6	7.10, d, 1.7	104.5	7.02, d, 2.3	104.0	7.09, d, 2.3	103.8	7.10, brs	95.7
7		165.1		164.6		163.8		162.0
8	6.90, d, 1.7	102.6	6.98, d, 2.3	104.2	6.93, d, 2.3	104.5	6.99, brs	103.7
9		162.2		161.4		161.4		163.5
9a		111.3		112.7		113.6		113.2
10		177.3		178.4		179.7		160.9
10a		157.1		129.1		132.7		109.0
1′	2.15, s	29.6	2.39, s	21.2	2.06, s	20.5	1.57, s	22.2
2′	-	-	3.56, s	44.6	-	-	-	-
7-Ome	3.93, s	56.1	3.96, s	56.2	3.92, s	55.9	3.95, s	56.3
9-Ome	3.93, s	56.6	3.91, s	56.5	3.88, s	56.4	3.98, s	56.8

**Table 2:** ED_50_ of cytotoxicity of compounds **1**–**5** in breast and pancreatic cancer cells.

Compound	MCF-7 (ED_50_ µM)	MIA PaCa-2 (ED_50_ µM)
**1**	2.9 [2.0–4.1] ^1^	>131
**2**	>69	>69
**3**	26.4 [24.3–28.7] ^1^	>52
**4**	4.0 [3.9–4.2] ^1^	>132
**5**	58.2 [53.7–63.1] ^1^	>126

^1^ 95% Confidence Intervals.

## Data Availability

Data presented in this study are available in Supplementary Material.

## Supplementary Materials

The following Supplementary Information is available online at https://www.mdpi.com/article/10.3390/molecules29020425/s1. Figure S1. UV (210, red, and 280, pink trace, nm) chromatogram of preparative RP-Flash Chromatography of a 3 L extract of the strain fermentation, in a 6 min step-wise gradient (blue trace) of acetone in water from 10% to 100% of acetone in 52 min. Fractions combined according to similar activity potency profile indicated as POOL 1 and 2. Figure S2. LC-UV (210, blue, and 280, orange trace, nm) chromatogram of POOL1 purification by preparative RP-HPLC applying a linear H_2_O:CH_3_CN gradient (CH_3_CN: 20–30%: 1–76 min, 100%: 77–90 min). Both solvents contain 0.1% TFA. Figure S3. HPLC 210 nm trace, UV-Vis, ((+)-ESI-TOF) and IR spectra of compound **1**. Figure S4. ^1^H NMR spectrum (DMSO-*d*_6_) of compound **1**. Figure S5. HSQC spectrum (DMSO-*d*_6_) of compound **1**. Figure S6. HPLC 210 nm trace, UV-Vis, ((+)-ESI-TOF) and IR spectra of compound **2**. Figure S7. ^1^H NMR spectrum (DMSO-*d*_6_) of compound **2**. Figure S8. ^13^C NMR spectrum (DMSO-*d*_6_) of compound **2**. Figure S9. HSQC (DMSO-*d*_6_) of compound **2**. Figure S10. HMBC (DMSO-*d*_6_) of compound **2**. Figure S11. HPLC 210 nm trace, UV-Vis, ((+)-ESI-TOF) and IR spectra of compound **3**. Figure S12. ^1^H NMR spectrum (DMSO-*d*_6_) of compound **3**. Figure S13. ^13^C NMR spectrum (DMSO-*d*_6_) of compound **3**. Figure S14. HSQC (DMSO-*d*_6_) of compound **3**. Figure S15. HMBC (DMSO-*d*_6_) of compound **3**. Figure S16. HPLC 210 nm trace, UV-Vis, ((+)-ESI-TOF) and IR spectra of compound **4**. Figure S17. ^1^H NMR spectrum (DMSO-*d*_6_) of compound **4**. Figure S18. ^13^C NMR spectrum (DMSO-*d*_6_) of compound **4**. Figure S19. HSQC (DMSO-*d*_6_) of compound **4**. Figure S20. HMBC (DMSO-*d*_6_) of compound **4** HPLC 210 nm trace, UV-Vis, ((+)-ESI-TOF) and IR spectra of compound **5**. Figure S22. ^1^H NMR spectrum (DMSO-*d*_6_) of compound **5**. Figure S23. ^13^C NMR spectrum (DMSO-*d*_6_) of compound **5**. Figure S24. HSQC (DMSO-*d*_6_) of compound **5**. Figure S25. HMBC (DMSO-*d*_6_) of compound **5**.
