# Supplementary material for: Naphthoquinone Derivatives from Angustimassarina populi CF-097565 Display Anti-Tumour Activity in 3D Cultures of Breast Cancer Cells
Source: Molecules. 2024 Jan 15;29(2):425. doi: 10.3390/molecules29020425 (PMC10820301; doi:10.3390/molecules29020425)
Supplement: Supplementary file 1 [file molecules-29-00425-s001.zip › molecules-2769064-supplementary.pdf]

Supplementary Materials

Naphthoquinone Derivatives from *Angustimassarina populi* CF-097565 Display Anti-tumour Activity in 3D Cultures of Breast Cancer cells

Thomas A. Mackenzie[1], Fernando Reyes[1], Marta Martinez[1], Victor González-Menéndez[1], Isabel Sánchez[1], Olga Genilloud [1], Jose R. Tormo[1,] * and Maria C. Ramos[1,] *

[1]Fundación MEDINA, Av. Conocimiento 34, Health Sciences Technology Park, 18016 Granada, Spain
*Correspondence: ruben.tormo@medinaandalucia.es (J.R.T.); Carmen.ramos@medinaandalucia.es (M.C.R); Tel.: +34 958 993 965

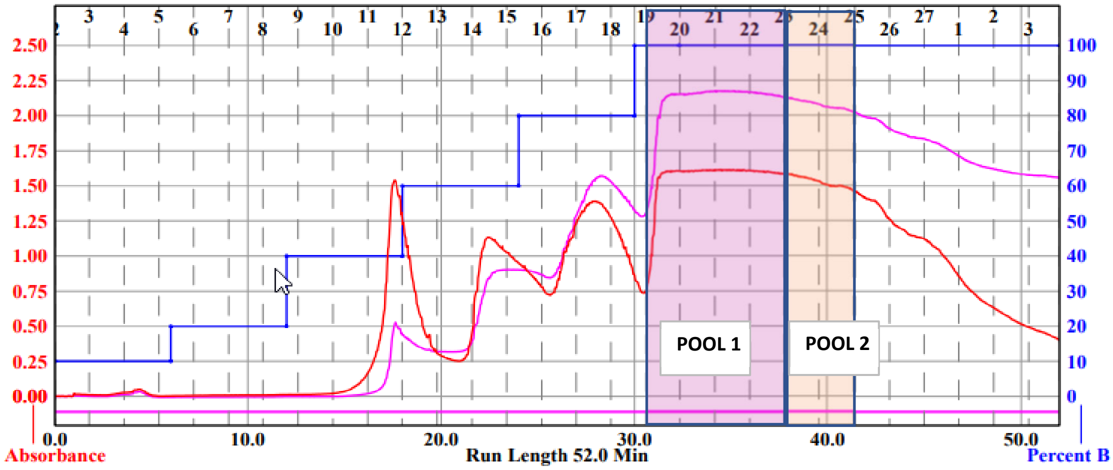

Figure S1. UV (210, red, and 280, pink trace, nm) chromatogram of preparative RP-Flash Chromatography of a 3L extract of the strain fermentation, in a stepped gradient (blue trace) of acetone in water from 10% to 100% of acetone in 52 min (6 min/step). Fractions combined according to similar bioactivity profile and chemical complexity are indicated as POOL 1 and 2.

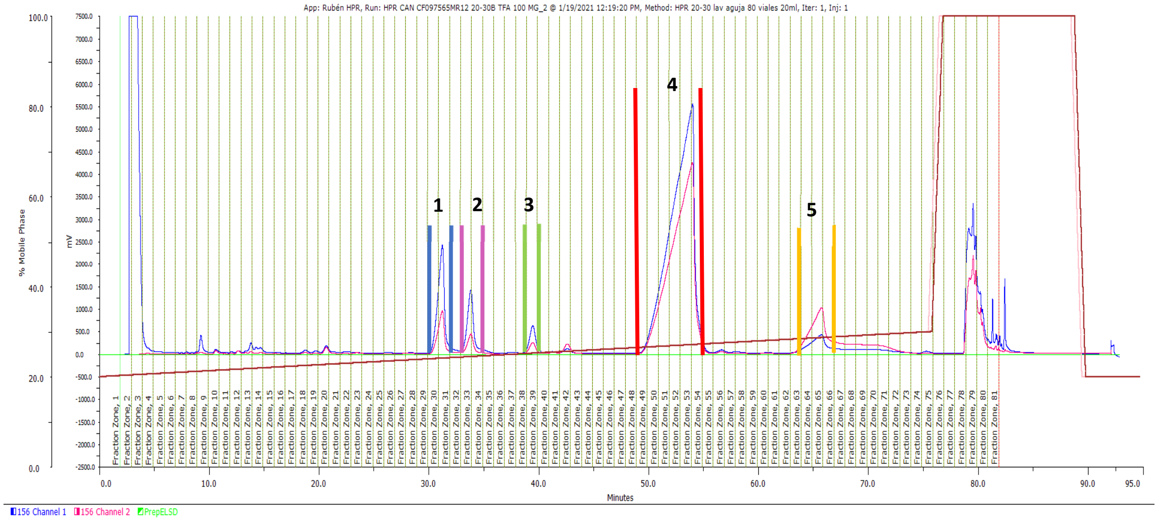

Figure S2. LC-UV (210, blue, and 280, orange trace, nm) chromatogram of POOL1 purification by preparative RP-HPLC applying a linear H_2O:CH_3CN gradient (CH_3CN: 20-30%: 1-76 min, 100%: 77-90 min). Both solvents contain 0.1% TFA. POOL2 behaved similarly.

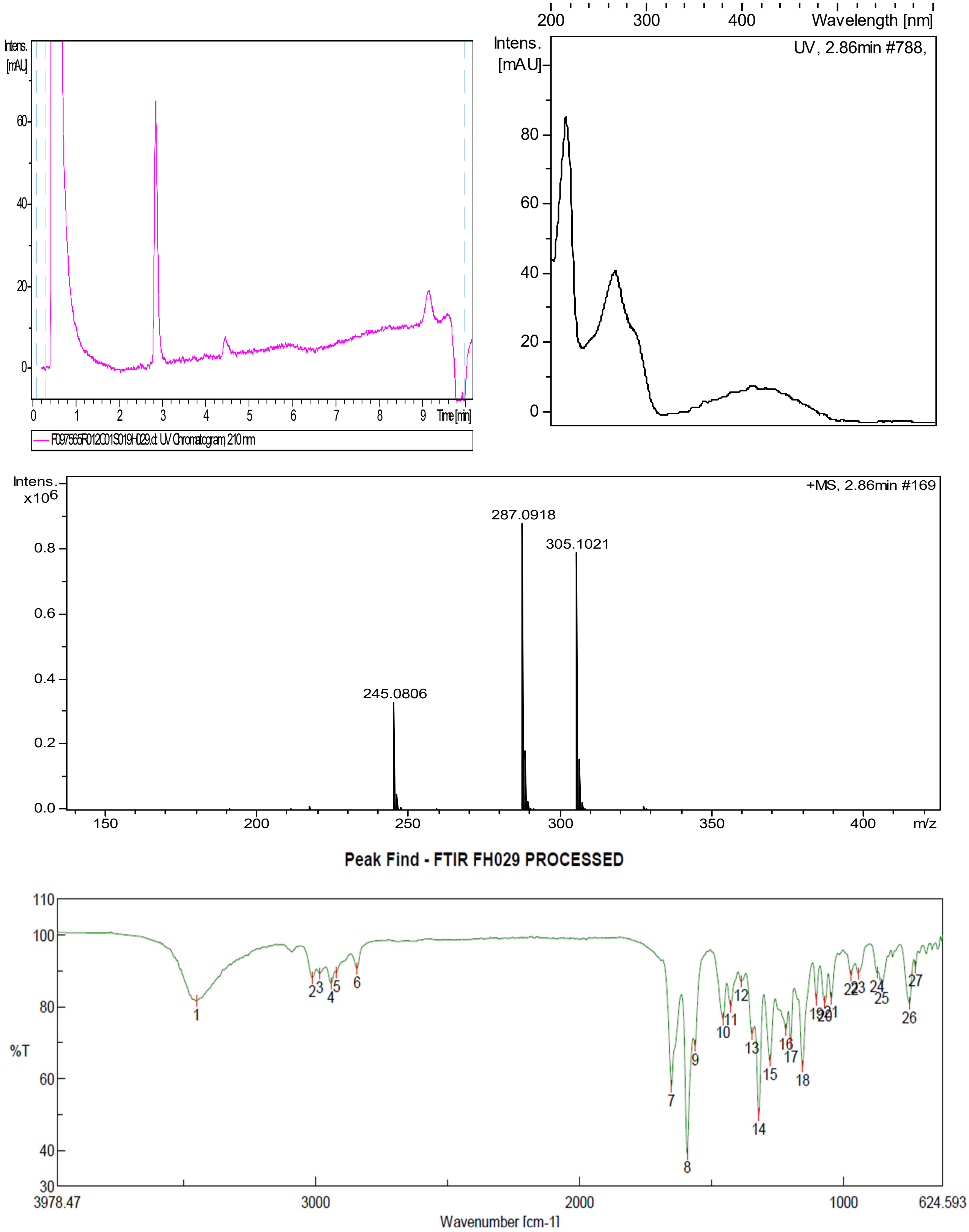

Figure S3. HPLC 210 nm trace, UV-Vis, ((+)-ESI-TOF) and IR spectra of compound **1** (purity 90% by UV).

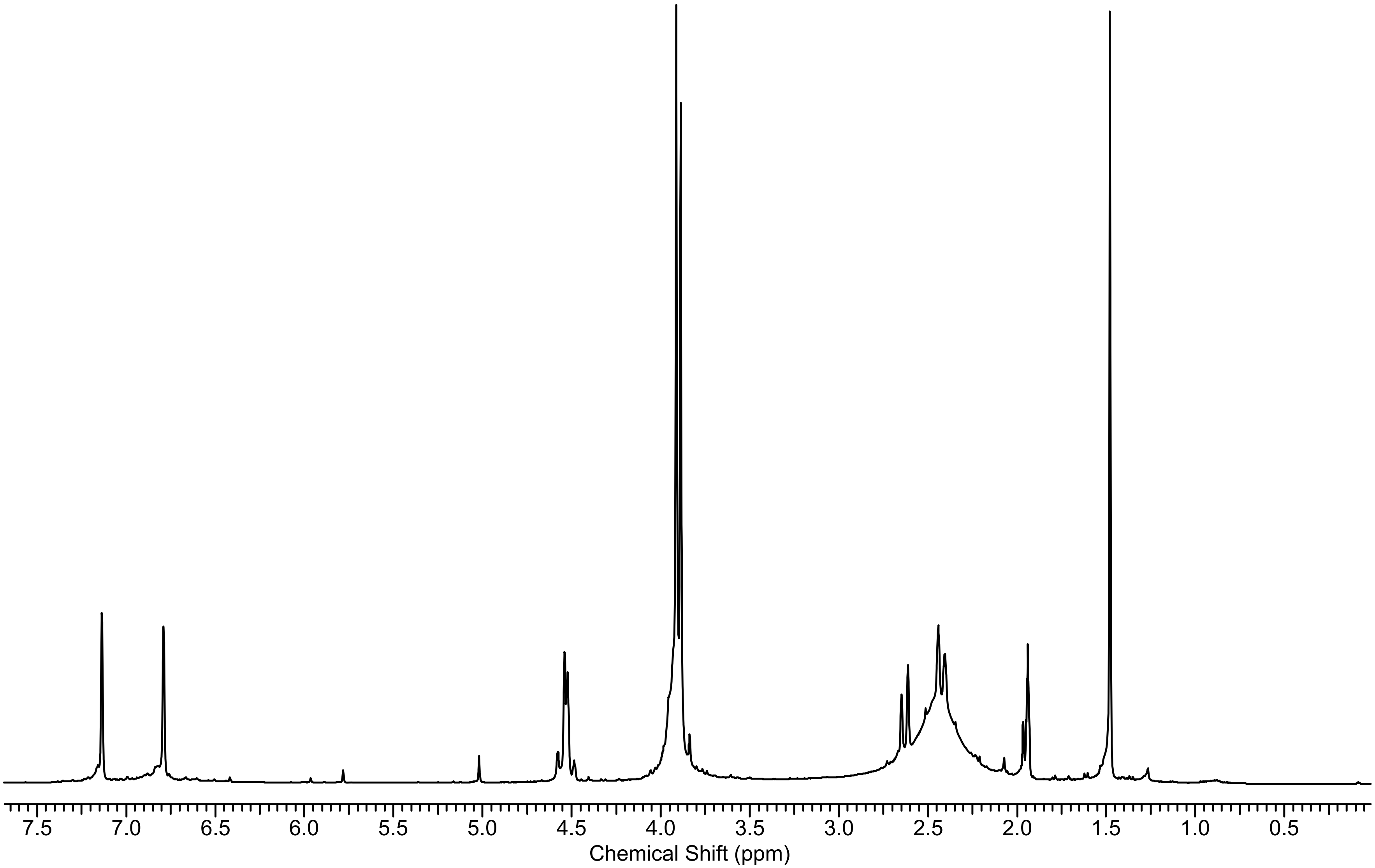

Figure S4. ^{1}H NMR spectrum (DMSO-d_6) of compound **1**.

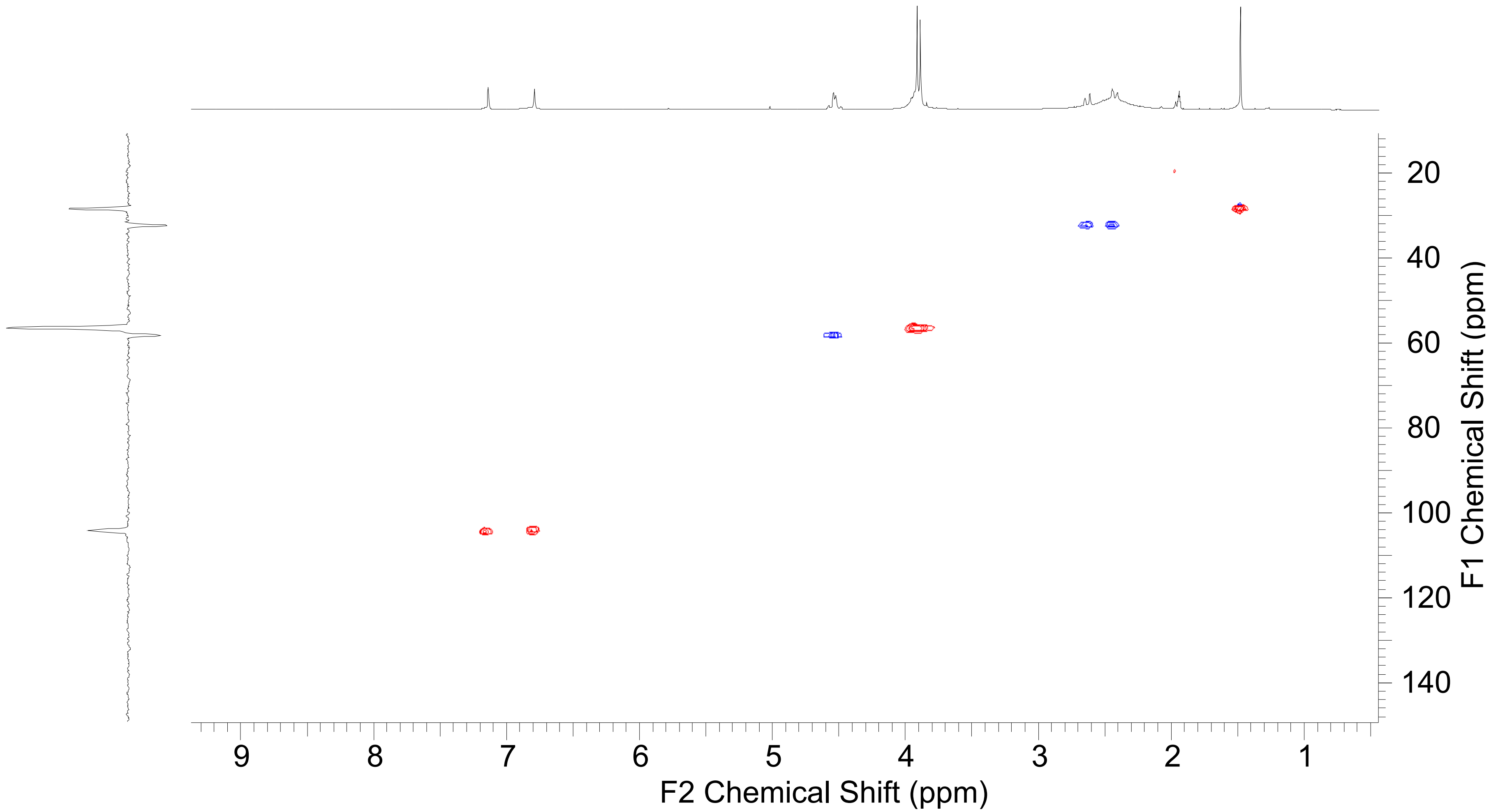

Figure S5. HSQC spectrum (DMSO-d_6) of compound **1**.

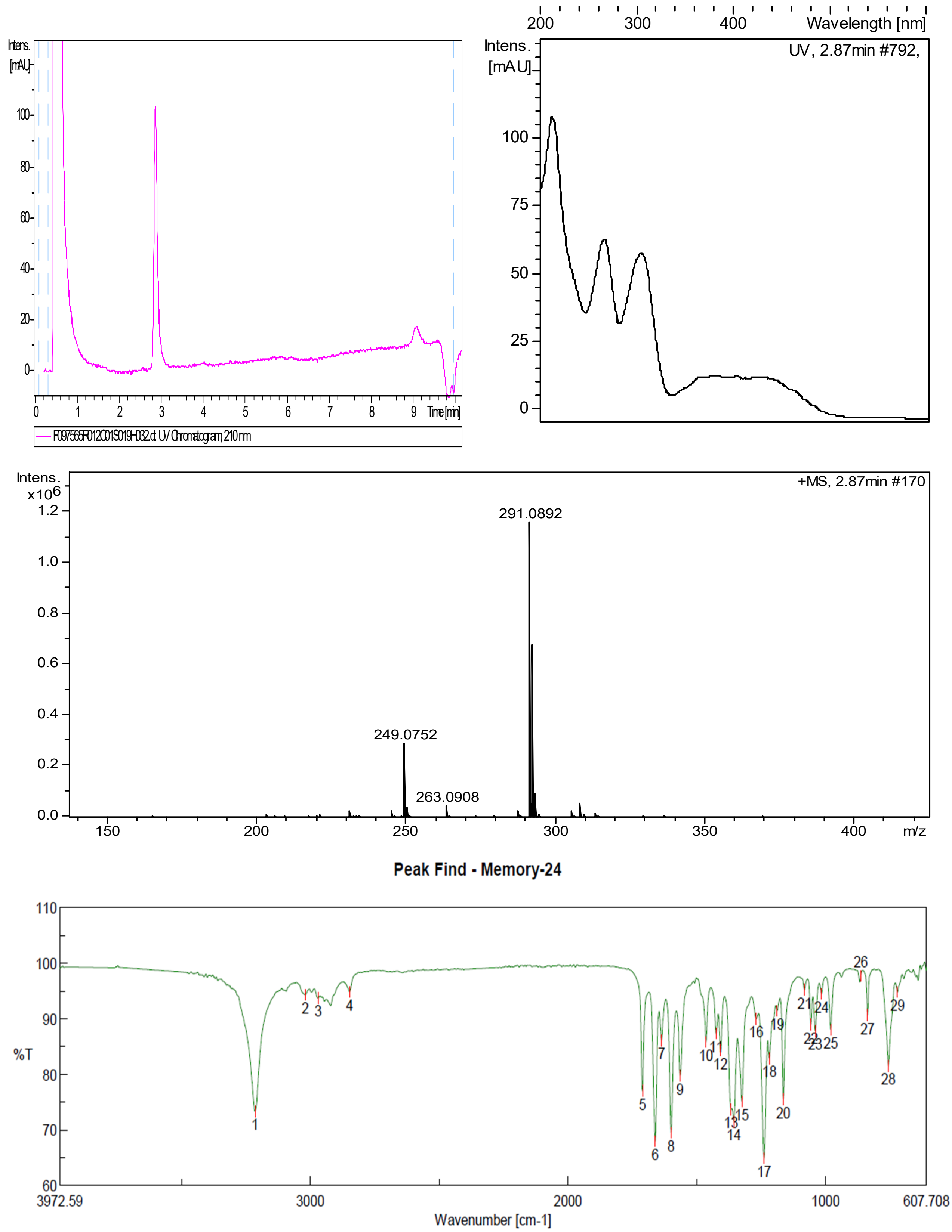

Figure S6. HPLC 210 nm trace, UV-Vis, ((+)-ESI-TOF) and IR spectra of compound **2** (purity 99% by UV).

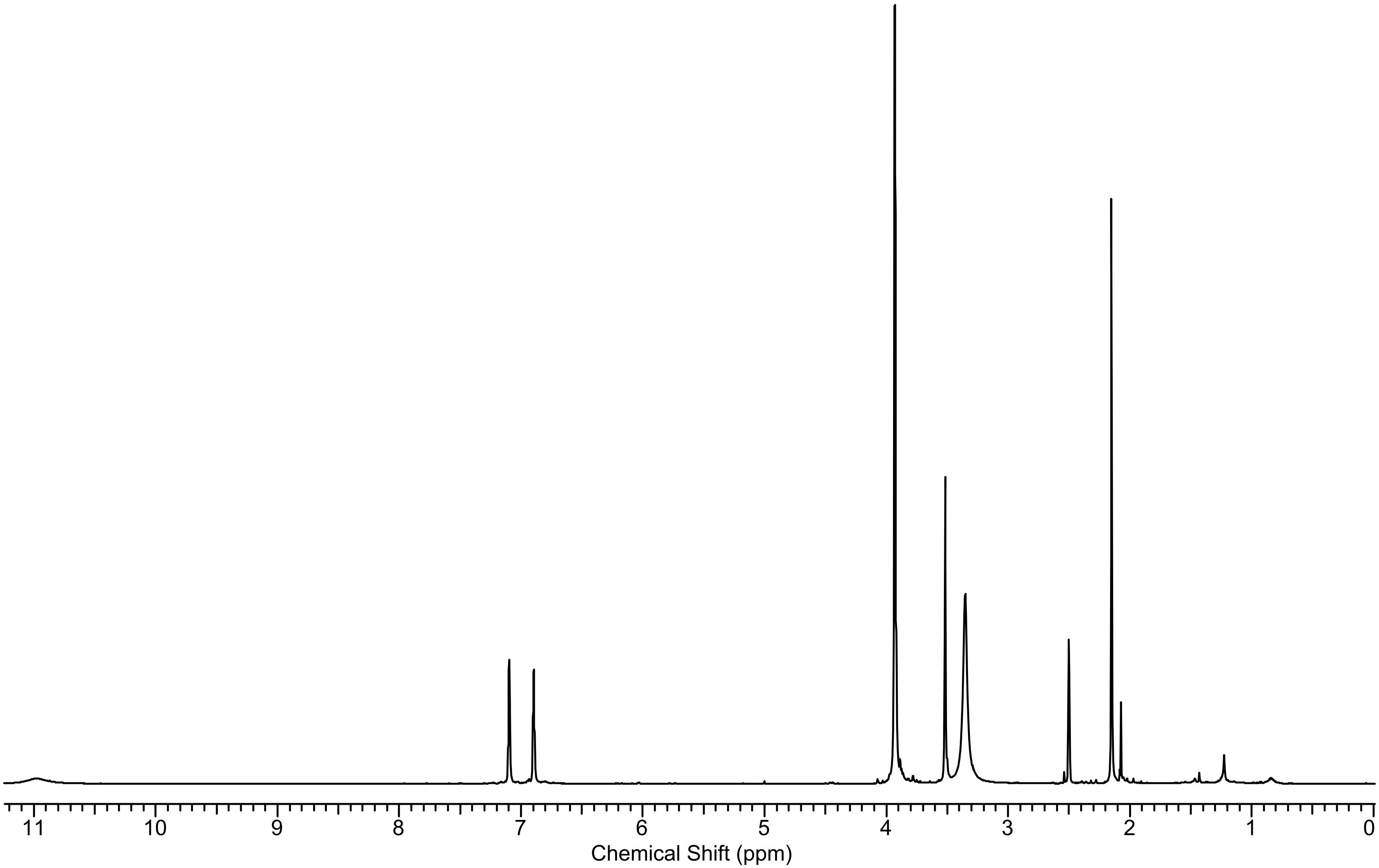

Figure S7. ^{1}H NMR spectrum (DMSO-d_6) of compound **2**.

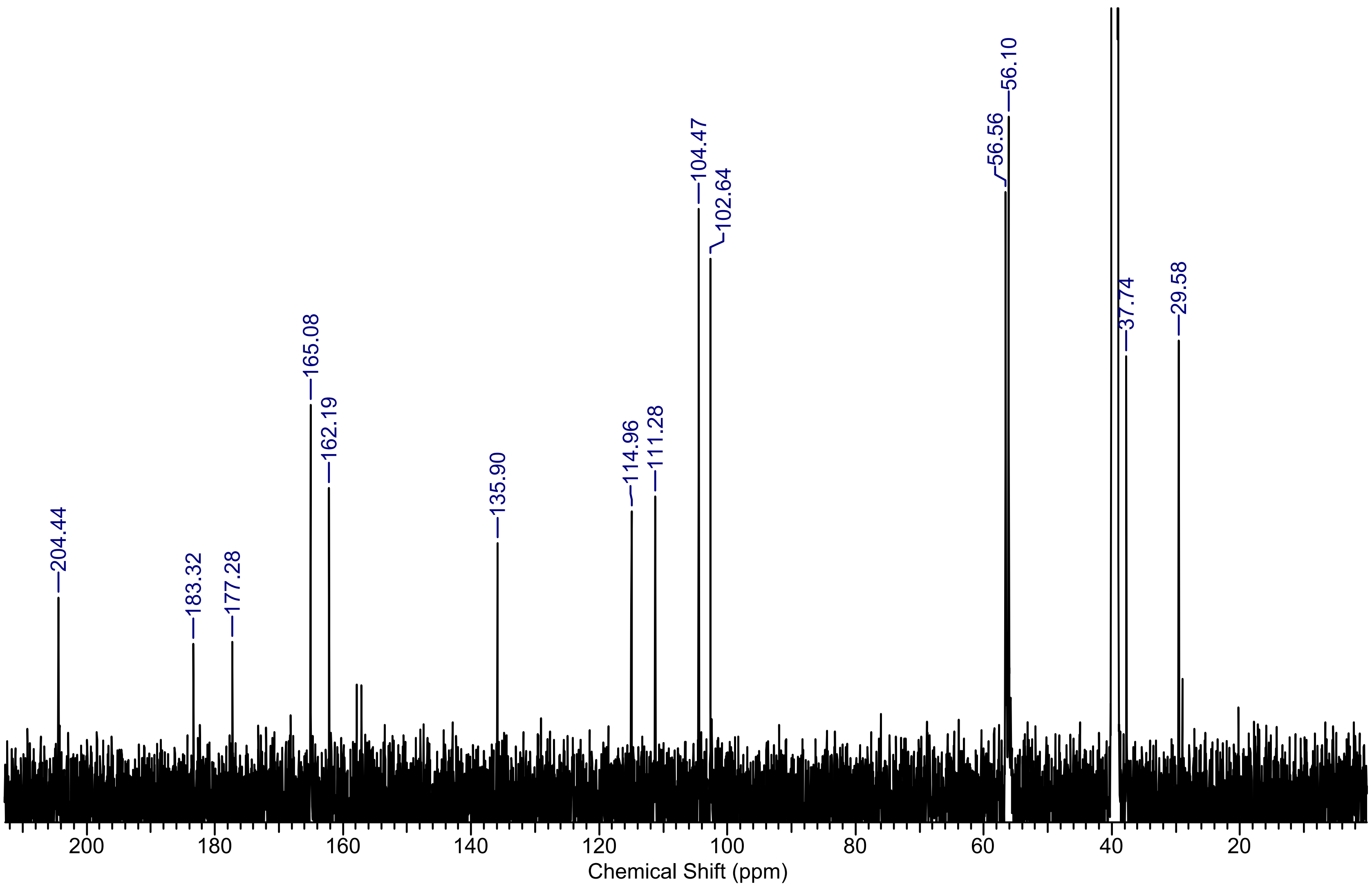

Figure S8. ^{13}C NMR spectrum (DMSO-d_6) of compound **2**.

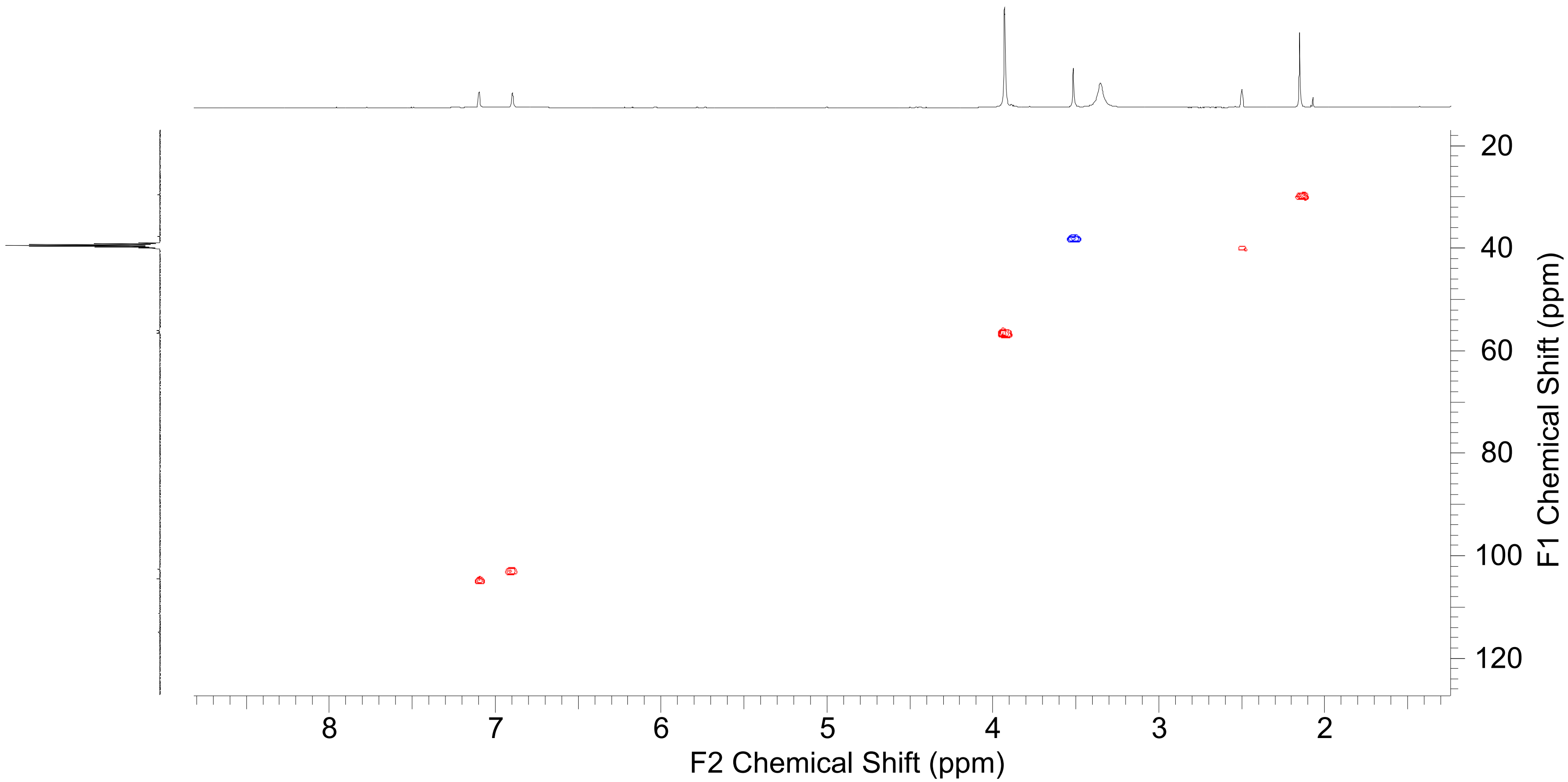

Figure S9. HSQC (DMSO-d_6) of compound **2**.

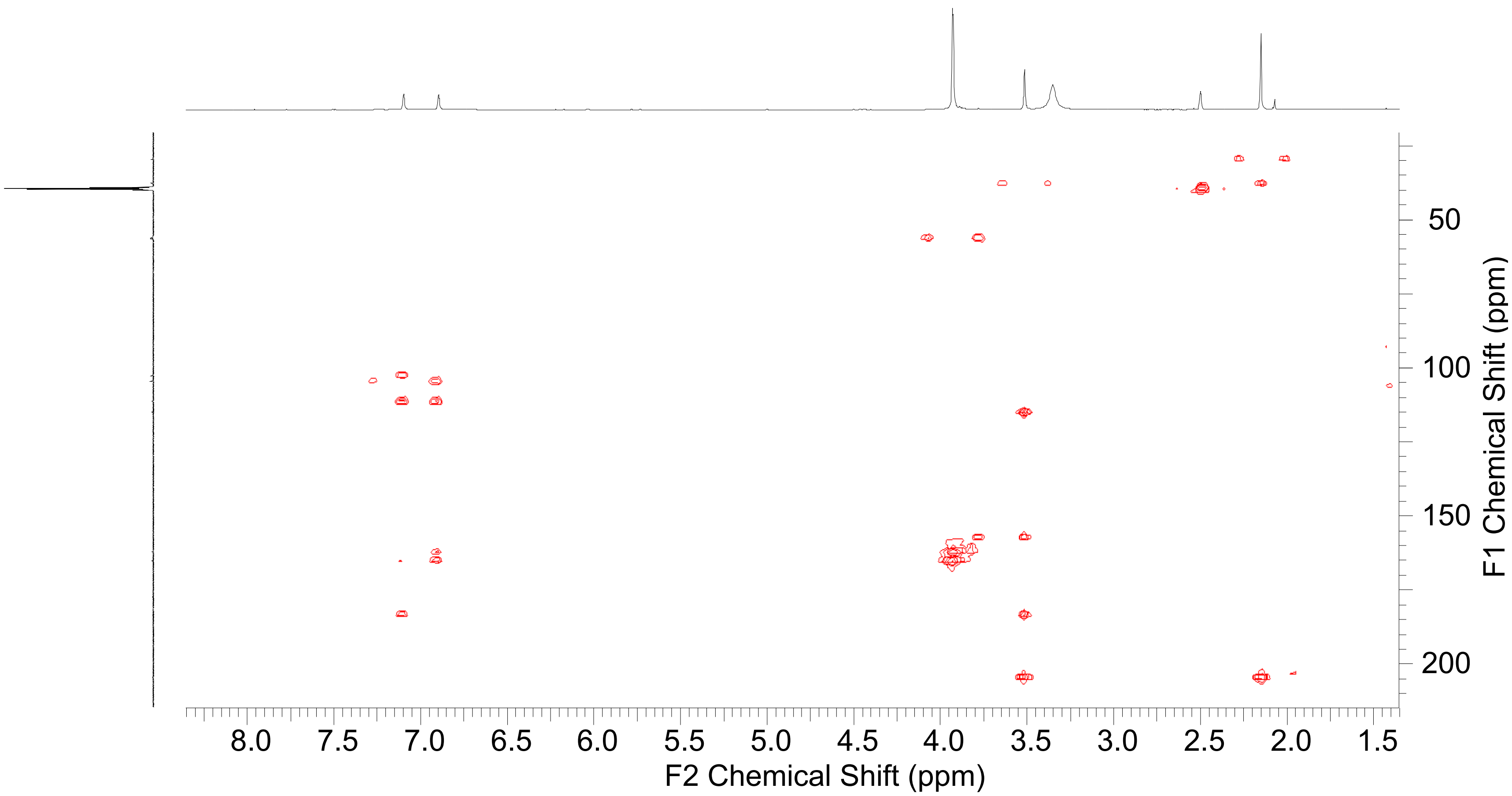

Figure S10. HMBC (DMSO-d_6) of compound **2**.

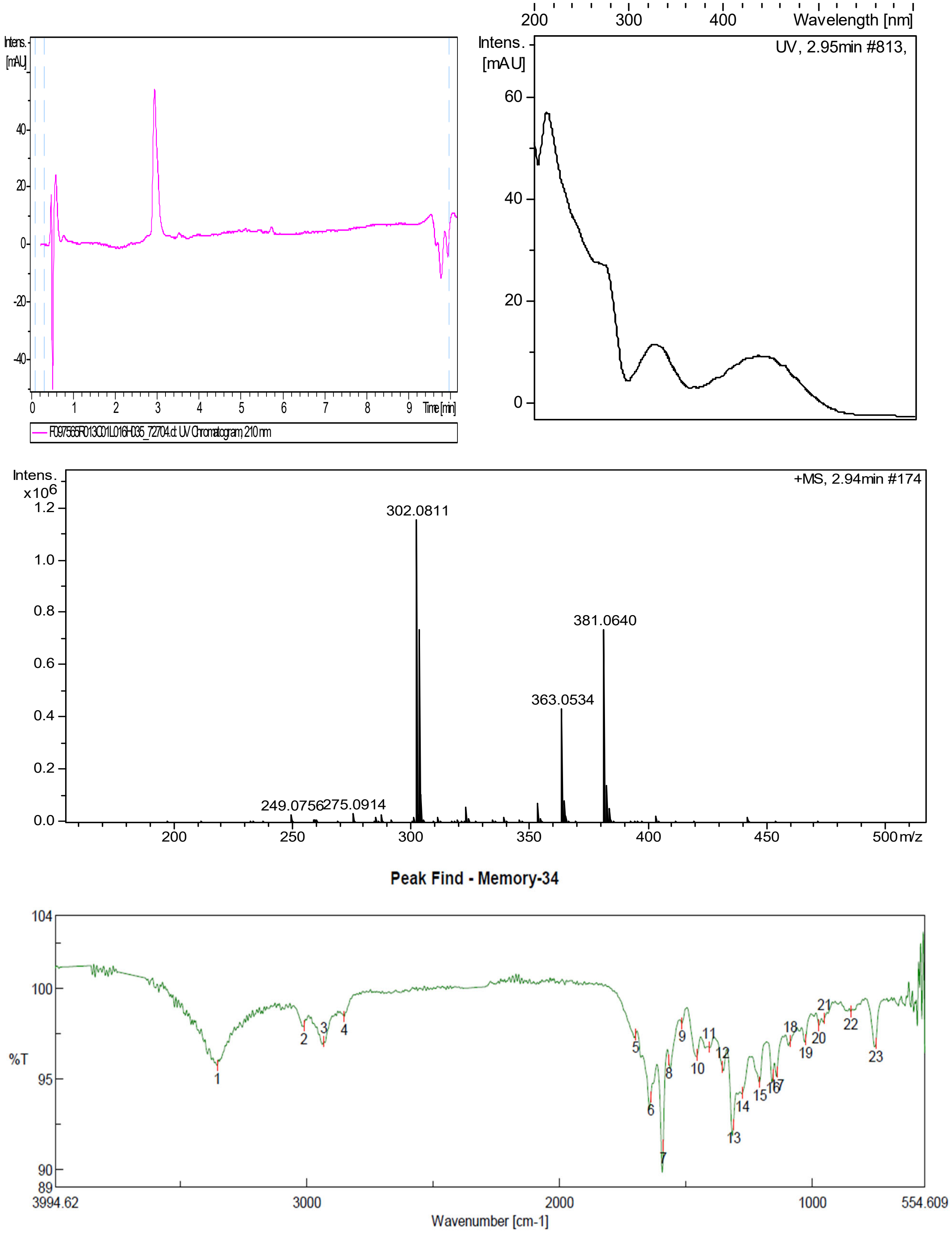

Figure S11. HPLC 210 nm trace, UV-Vis, ((+)-ESI-TOF) and IR spectra of compound **3** (purity 95% by UV).

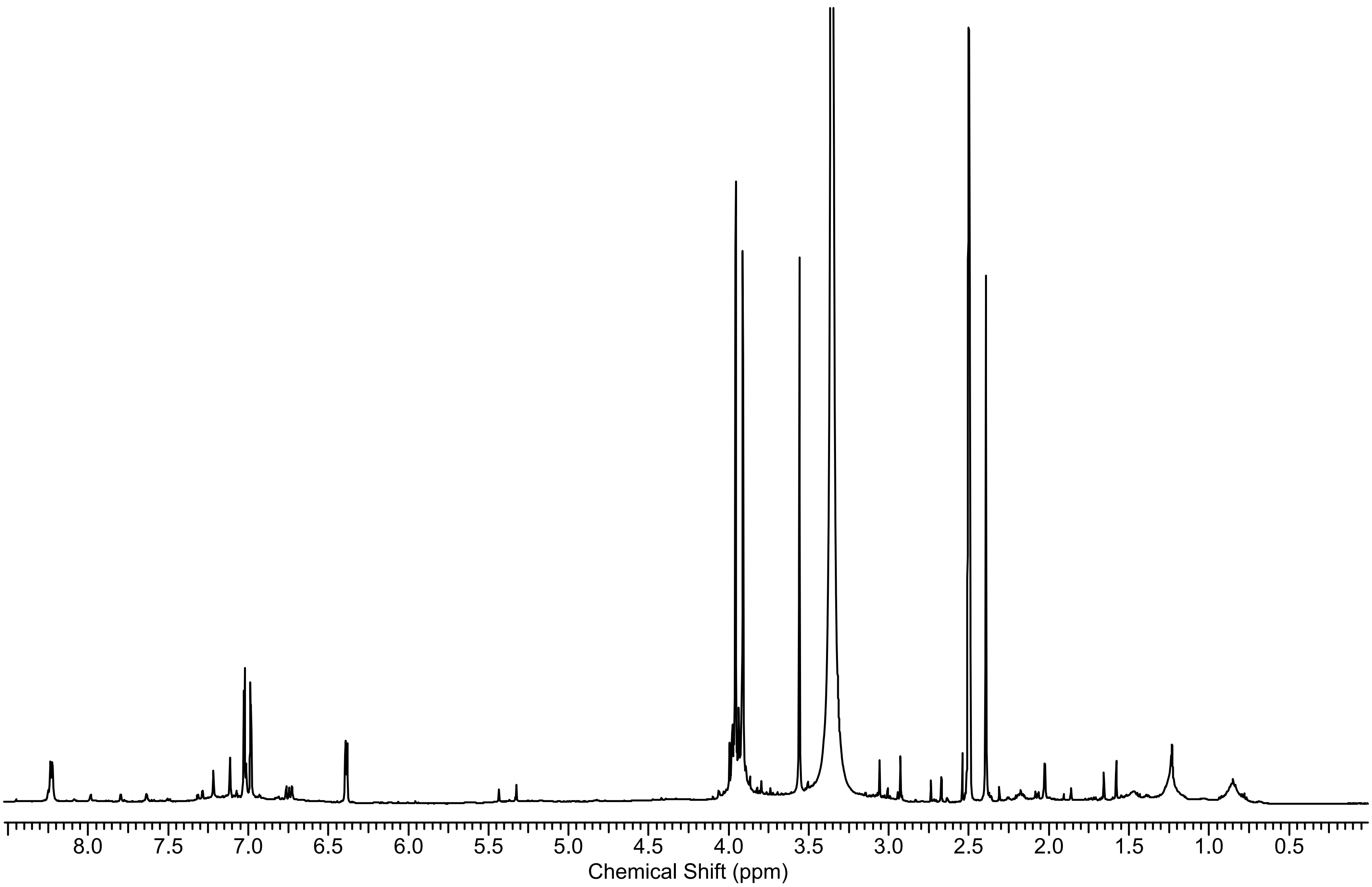

Figure S12. ^{1}H NMR spectrum (DMSO-d_6) of compound **3**.

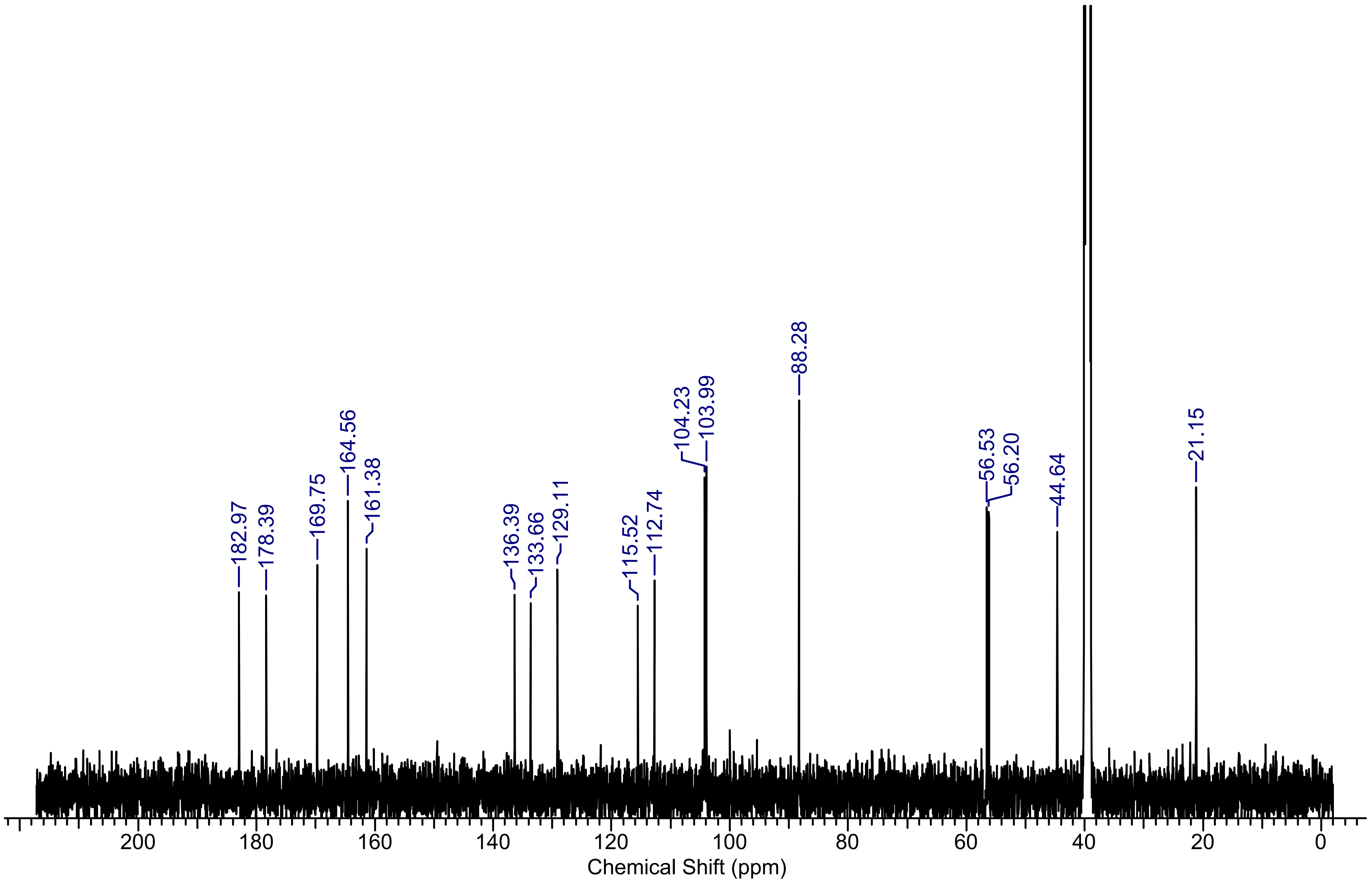

Figure S13. ^{13}C NMR spectrum (DMSO-d_6) of compound **3**.

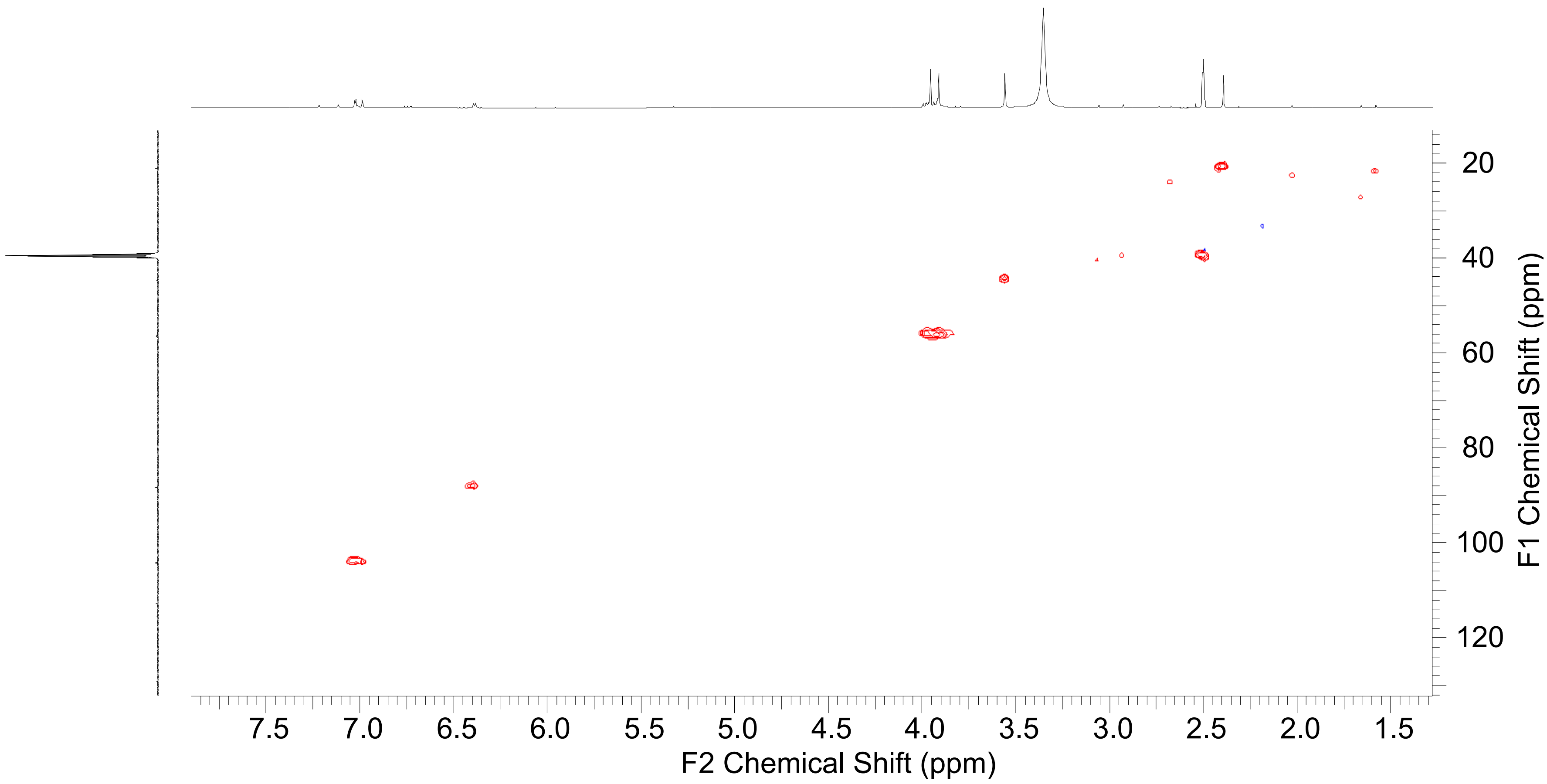

Figure S14. HSQC (DMSO-d_6) of compound **3**.

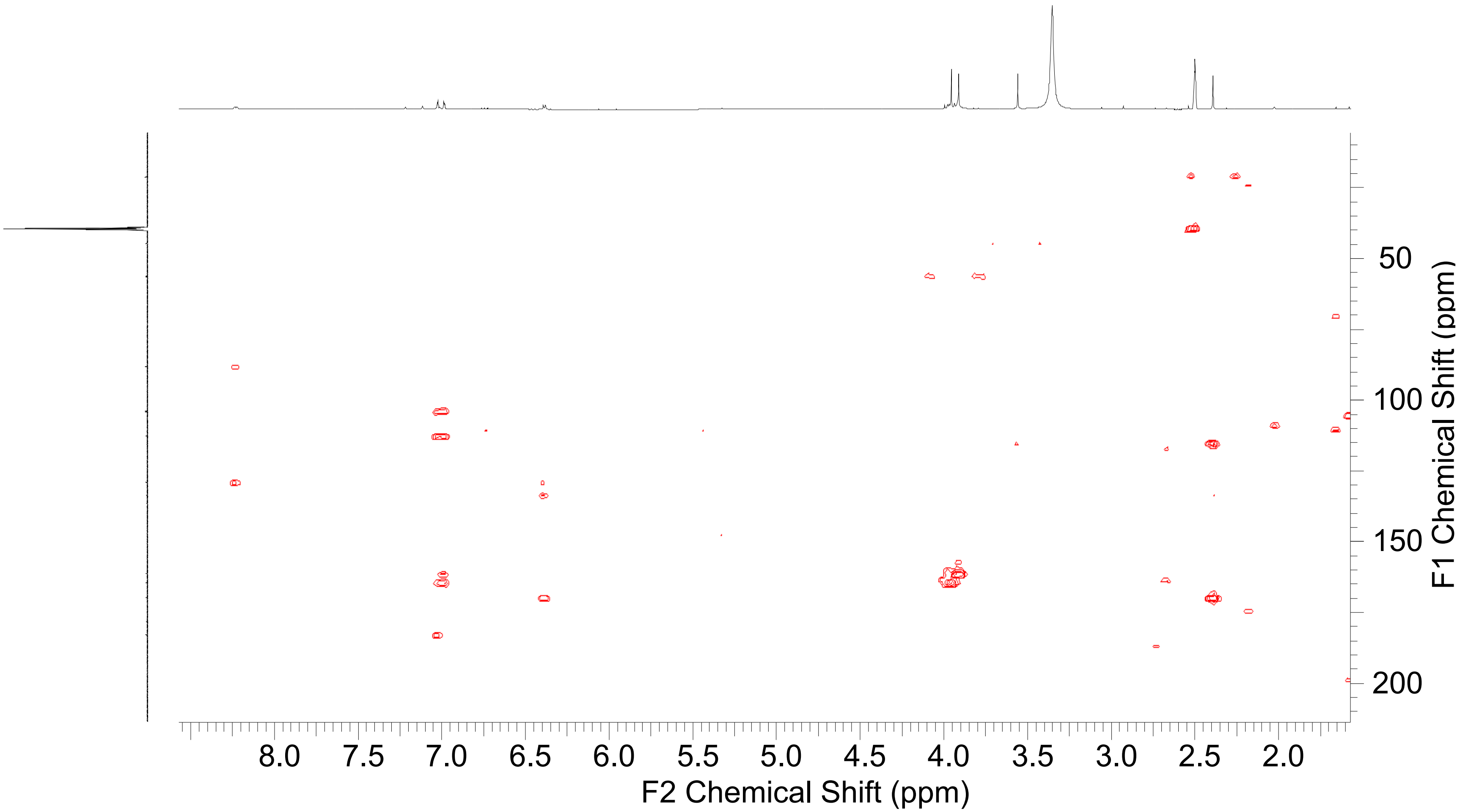

Figure S15. HMBC (DMSO-d_6) of compound **3**

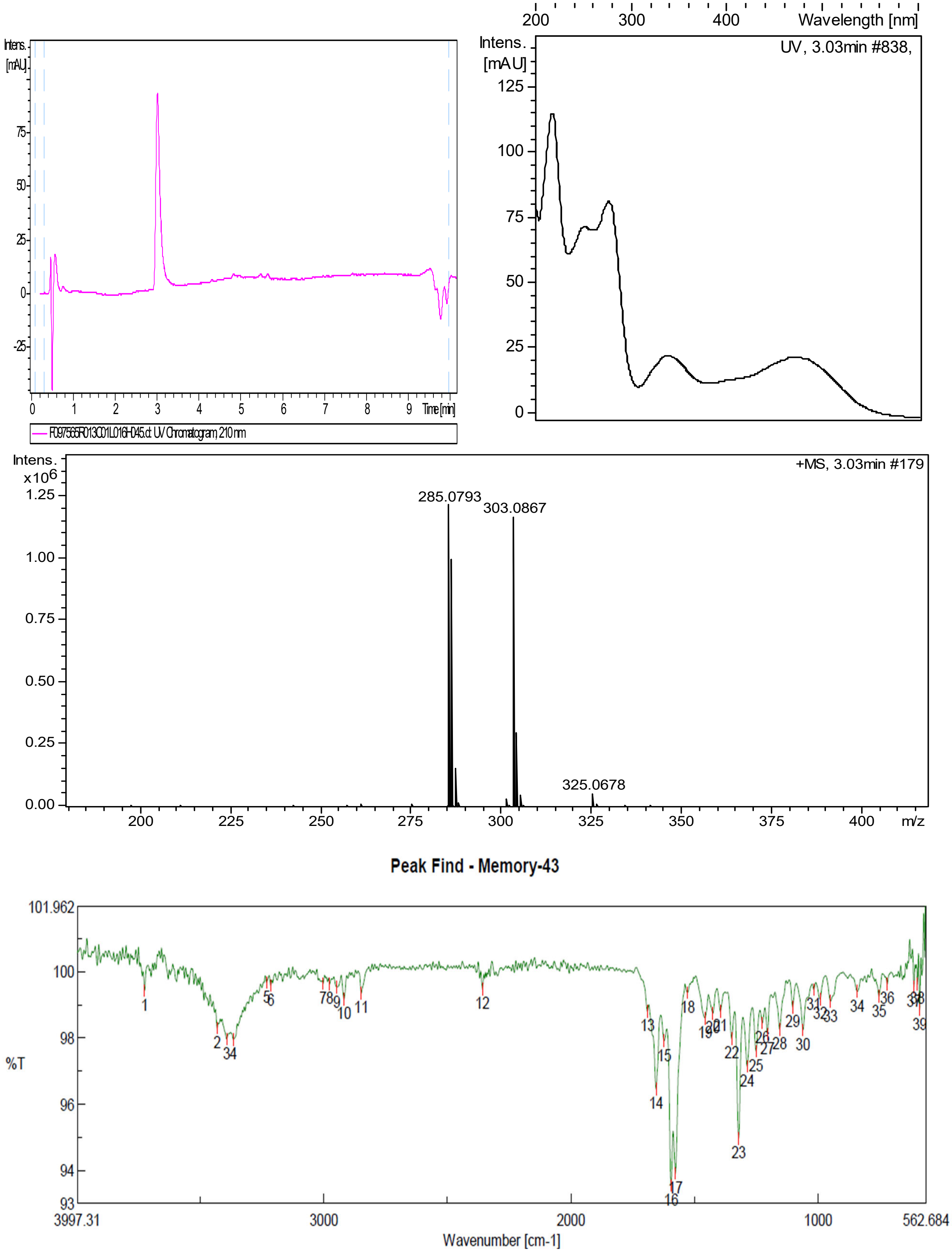

Figure S16. HPLC 210 nm trace, UV-Vis, ((+)-ESI-TOF) and IR spectra of compound **4** (purity 94% by UV).

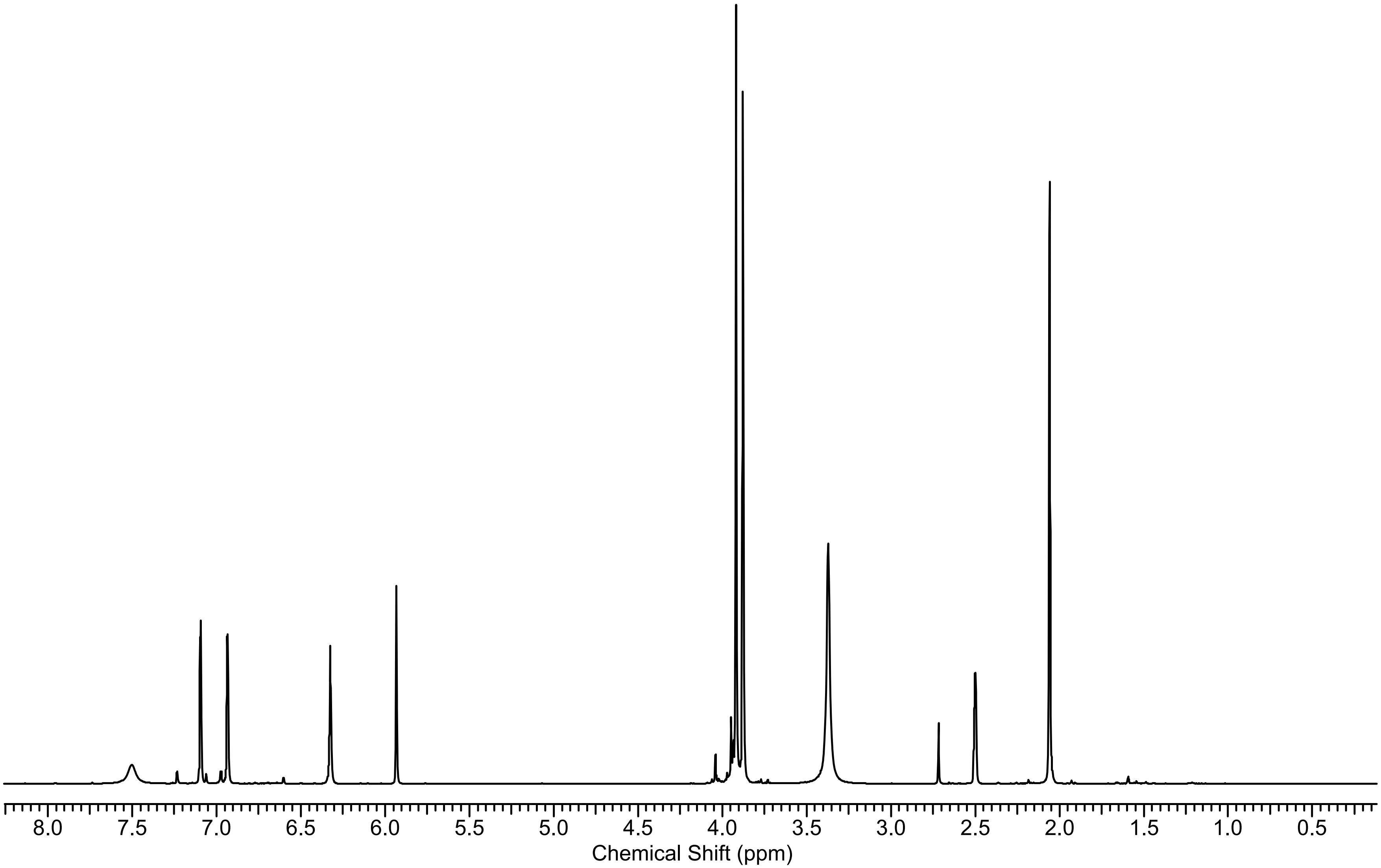

Figure S17. ^{1}H NMR spectrum (DMSO-d_6) of compound **4**.

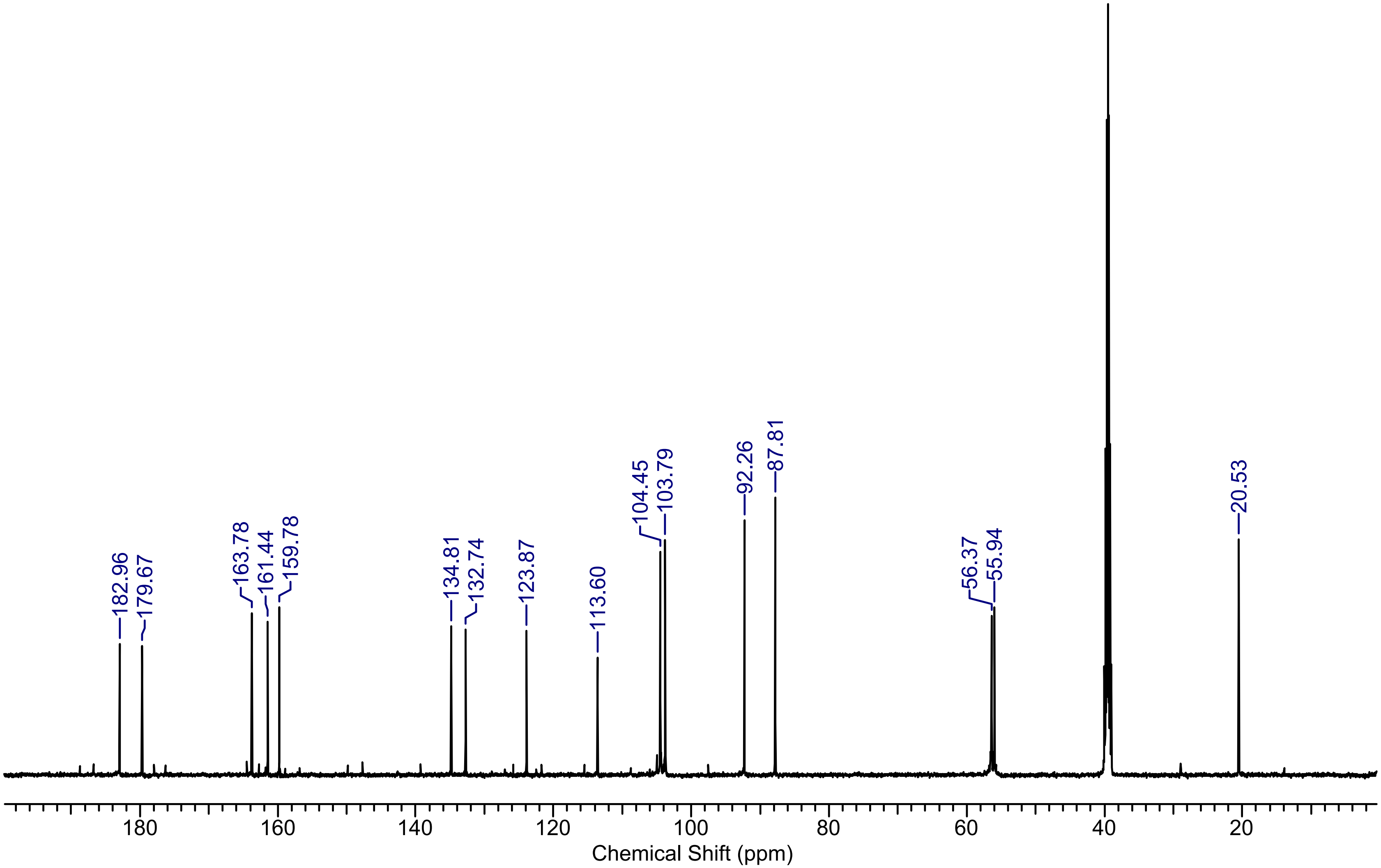

Figure S18. ^{13}C NMR spectrum (DMSO-d_6) of compound **4**.

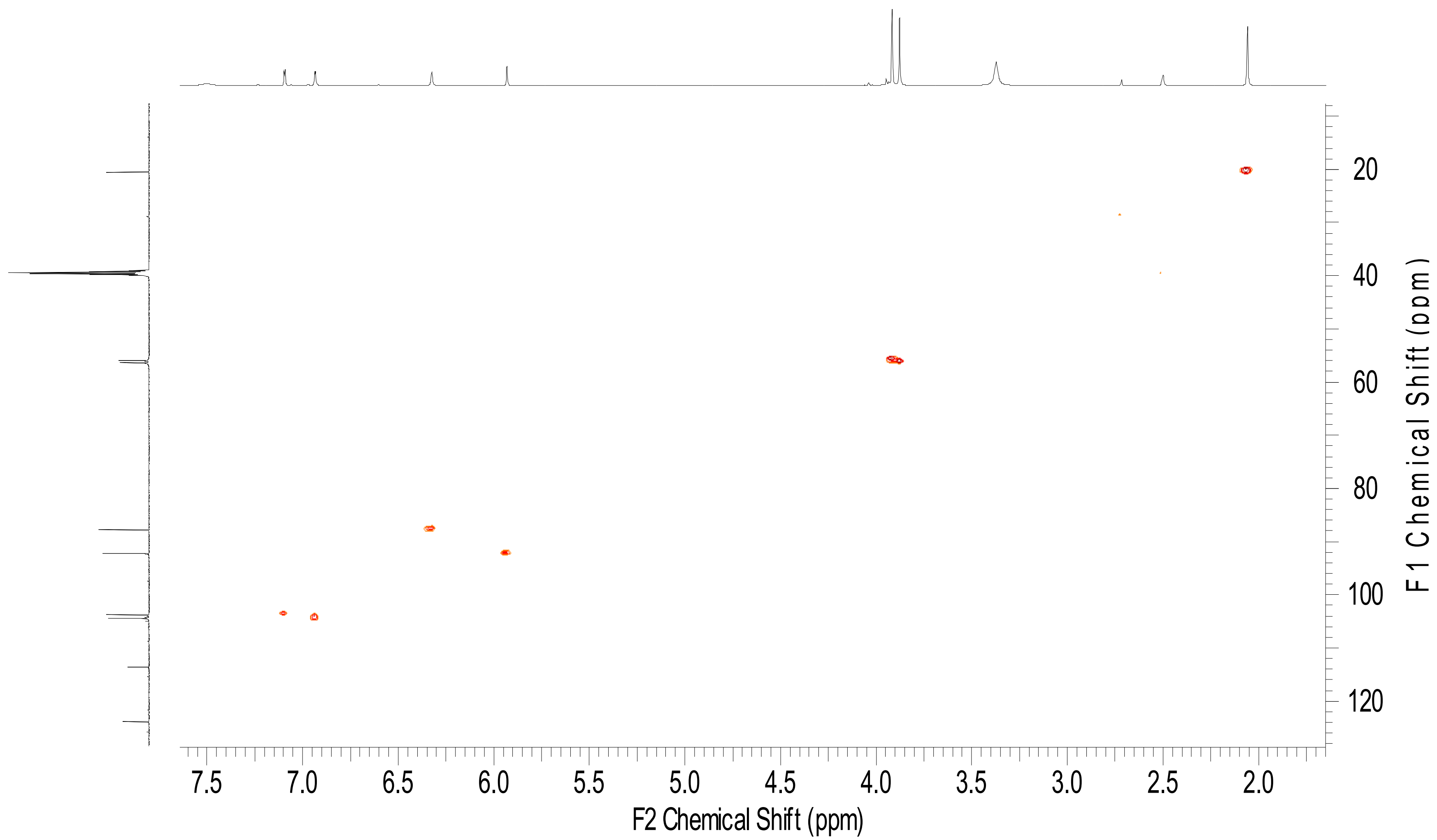

Figure S19. HSQC (DMSO-d_6) of compound **4**.

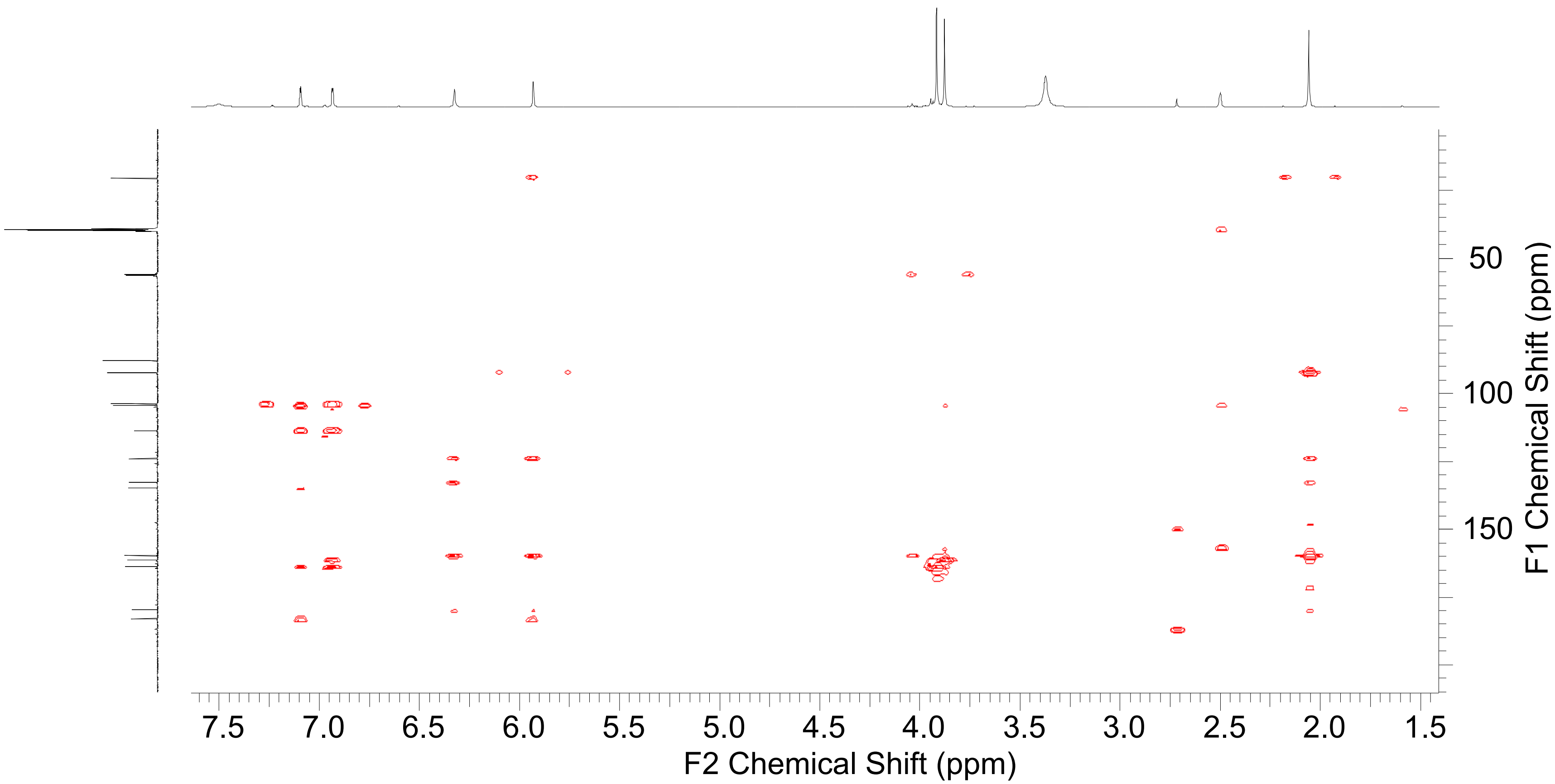

Figure S20. HMBC (DMSO-d_6) of compound **4**.

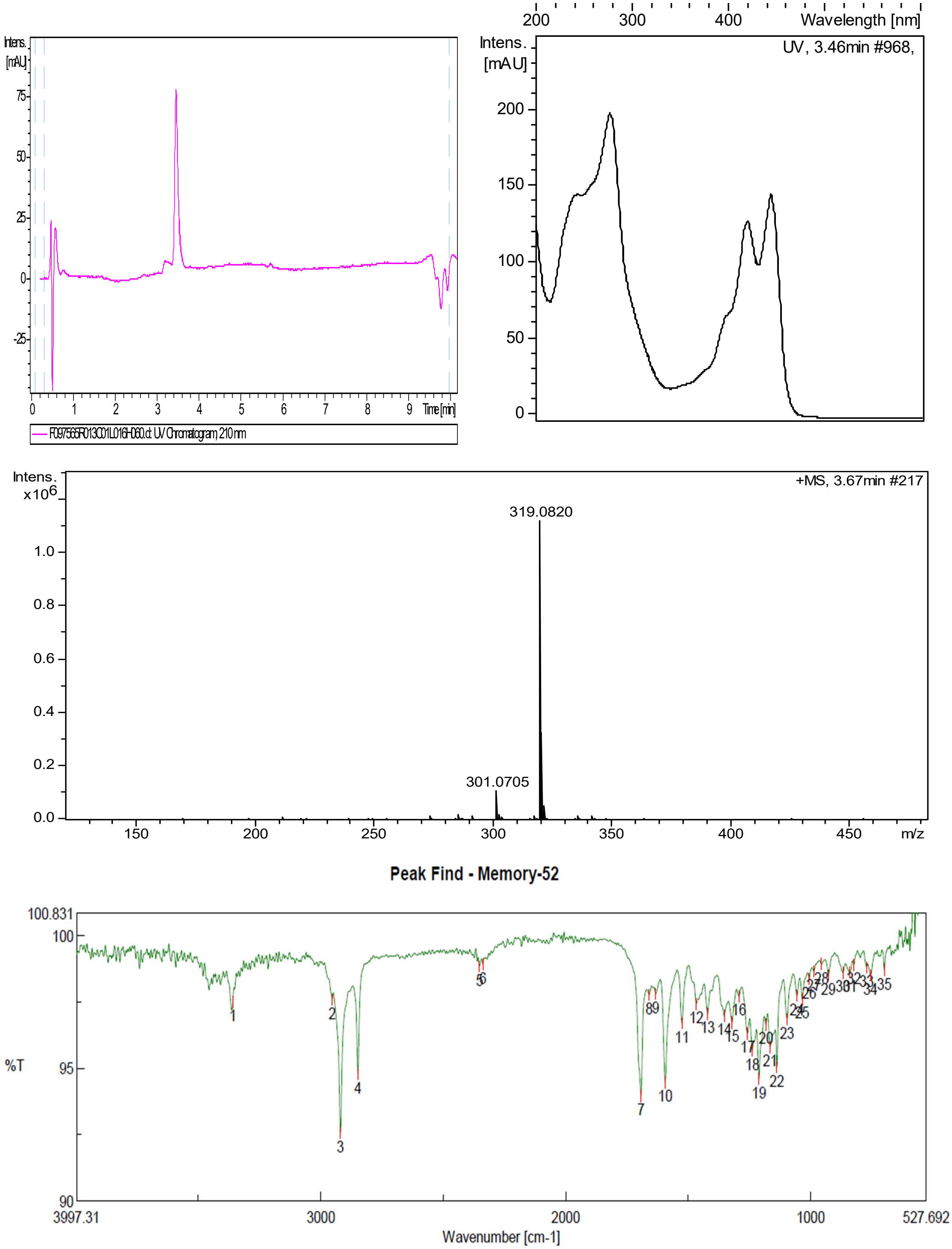

Figure S21. HPLC 210 nm trace, UV-Vis, ((+)-ESI-TOF) and IR spectra of compound **5** (purity 89% by UV).

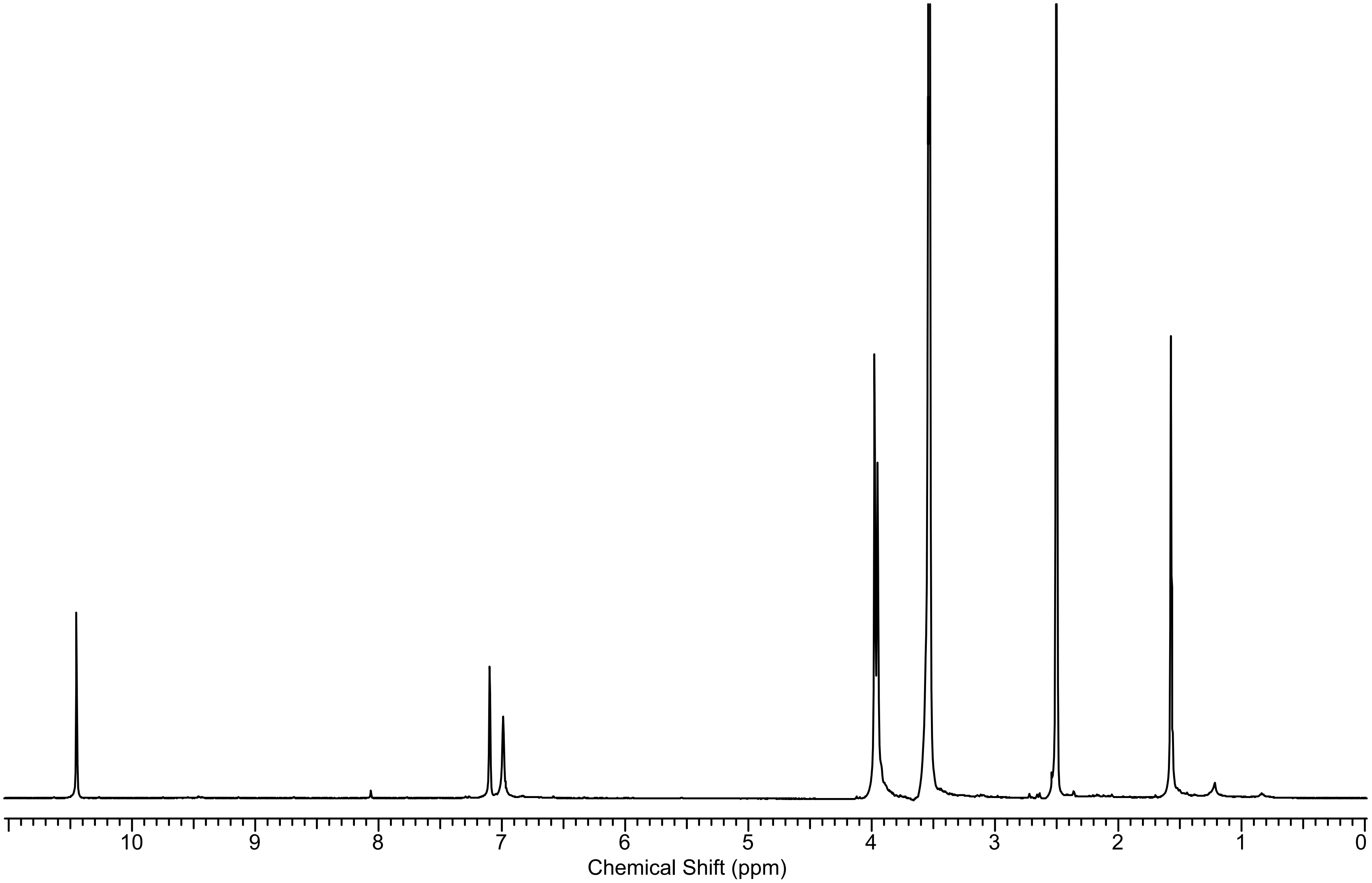

Figure S22. ^{1}H NMR spectrum (DMSO-d_6) of compound **5**.

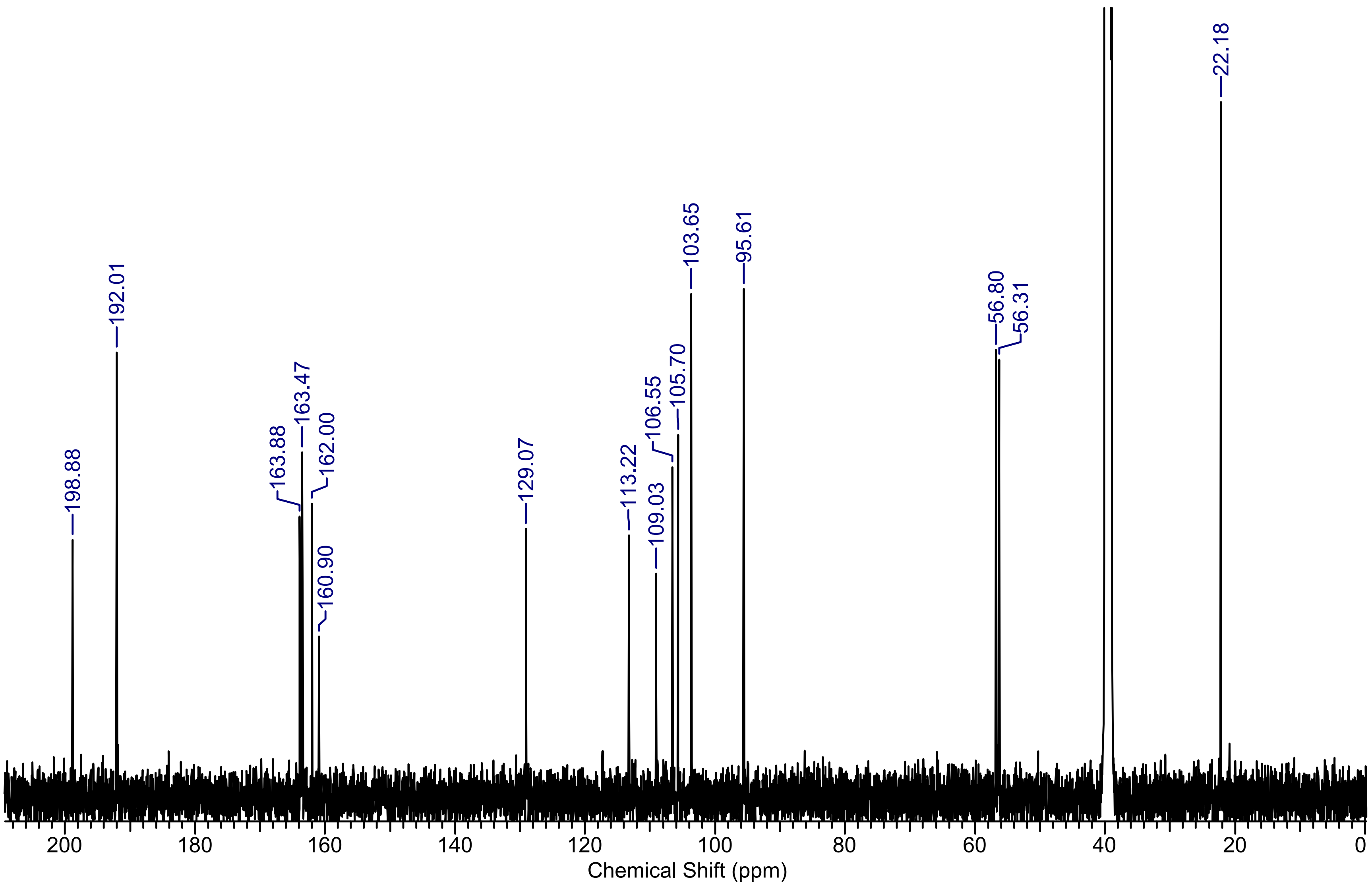

Figure S23. ^{13}C NMR spectrum (DMSO-d_6) of compound **5**.

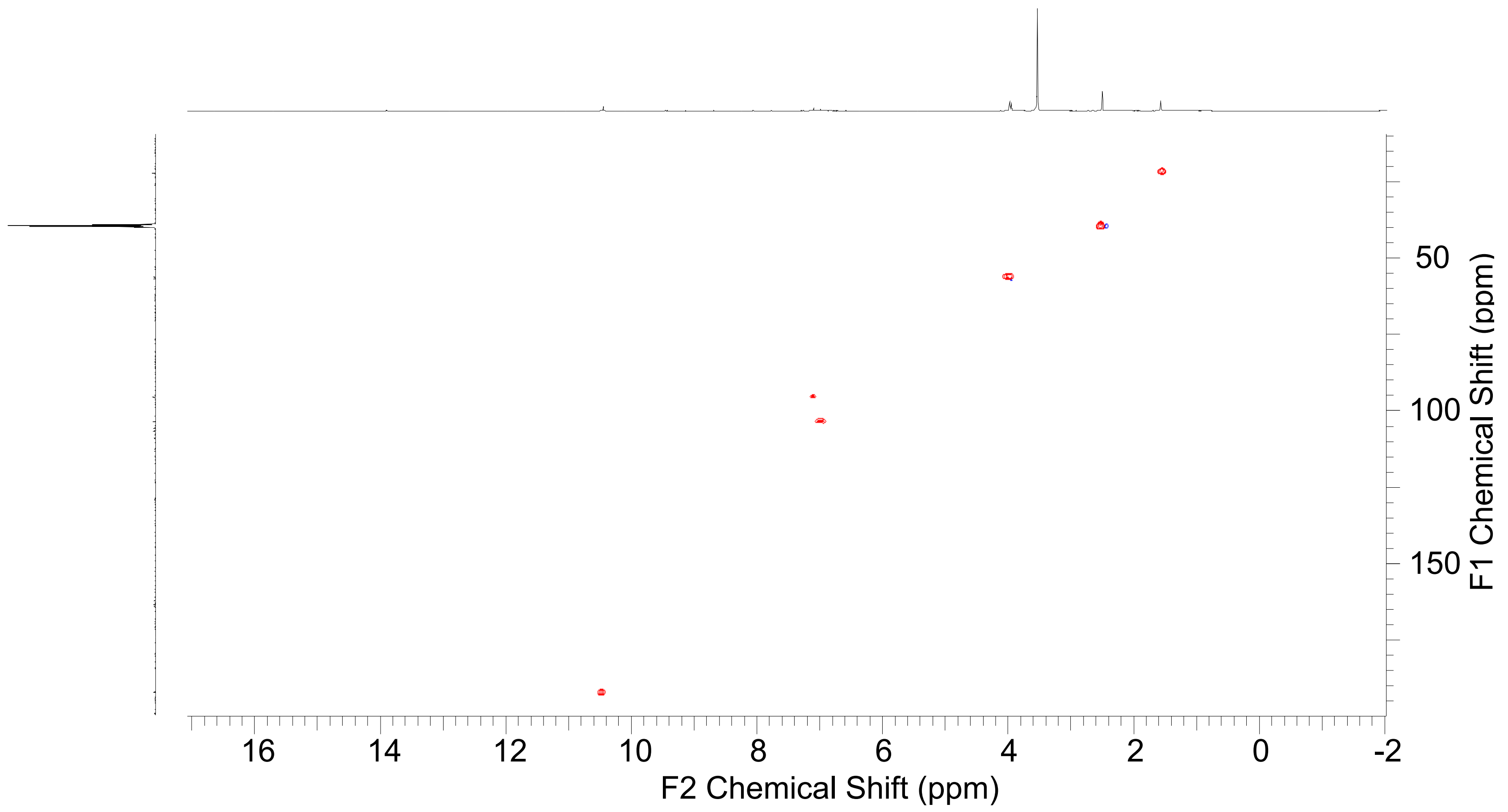

Figure S24. HSQC (DMSO-d_6) of compound **5**.

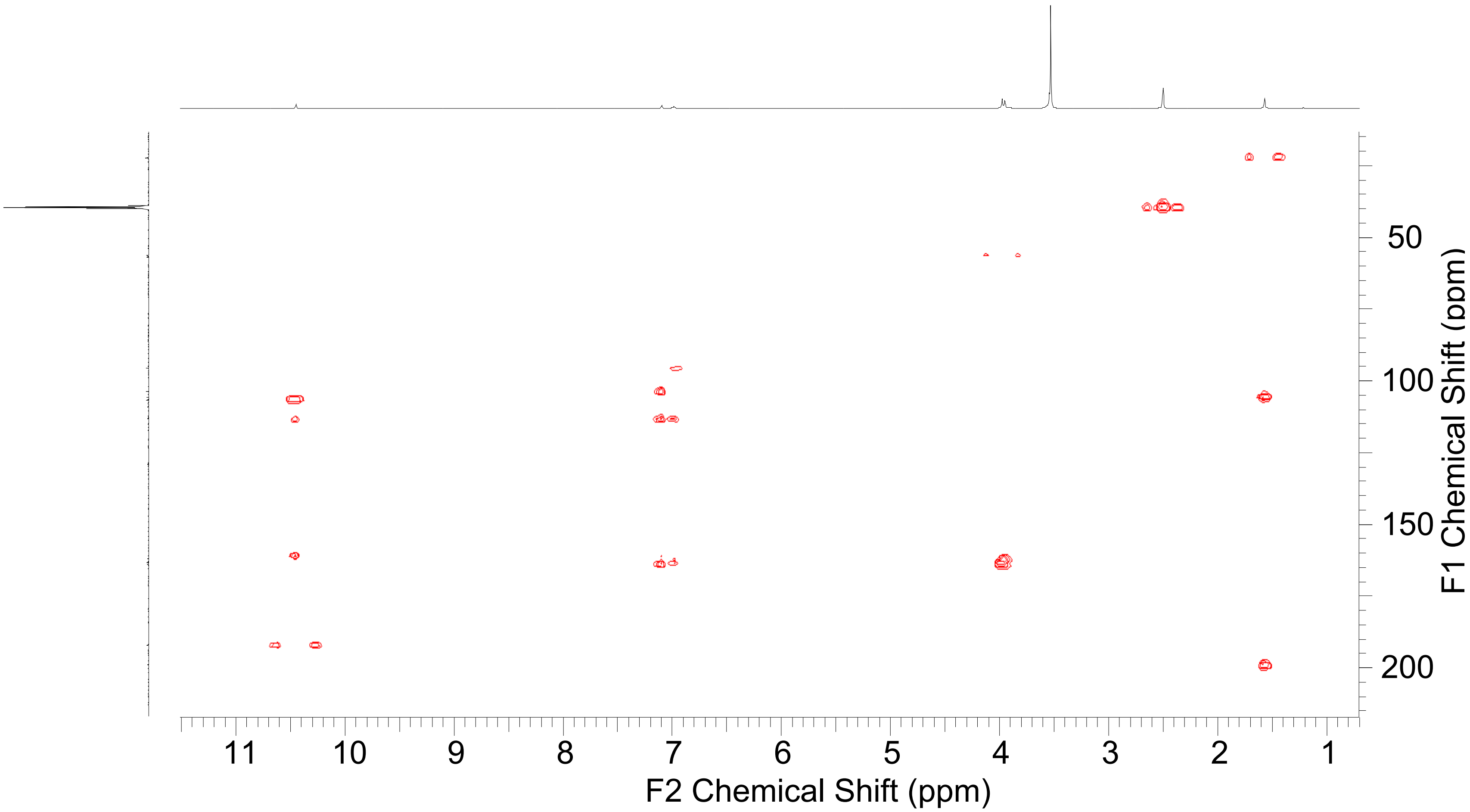

Figure S25. HMBC (DMSO-d_6) of compound **5**.
